# High dimensional model representation of log-likelihood ratio: binary classification with expression data

**DOI:** 10.1186/s12859-020-3486-x

**Published:** 2020-04-25

**Authors:** Ali Foroughi pour, Maciej Pietrzak, Lori A Dalton, Grzegorz A. Rempała

**Affiliations:** 1 Department of Electrical and Computer Engineering, The Ohio State University, 205 Dreese laboratories, 2015 Neil Ave., Columbus, 43210 USA; 2 Department of Mathematics, The Ohio State University, 100 Math Tower, 31 West 18th Ave., Columbus, 43210 USA; 3 Department of Biomedical Informatics, The Ohio State University, 1585 Neil Ave, Columbus, 43210 USA; 4 College of Public Health, 250 Cunz Hall, 1841 Neil Ave., Columbus, 43210 USA

**Keywords:** High dimensional model representation, Classification, Disease prediction, Log-likelihood ratio, Expression analysis

## Abstract

**Background:**

Binary classification rules based on a small-sample of high-dimensional data (for instance, gene expression data) are ubiquitous in modern bioinformatics. Constructing such classifiers is challenging due to (a) the complex nature of underlying biological traits, such as gene interactions, and (b) the need for highly interpretable glass-box models. We use the theory of high dimensional model representation (HDMR) to build interpretable low dimensional approximations of the log-likelihood ratio accounting for the effects of each individual gene as well as gene-gene interactions. We propose two algorithms approximating the second order HDMR expansion, and a hypothesis test based on the HDMR formulation to identify significantly dysregulated pairwise interactions. The theory is seen as flexible and requiring only a mild set of assumptions.

**Results:**

We apply our approach to gene expression data from both synthetic and real (breast and lung cancer) datasets comparing it also against several popular state-of-the-art methods. The analyses suggest the proposed algorithms can be used to obtain interpretable prediction rules with high prediction accuracies and to successfully extract significantly dysregulated gene-gene interactions from the data. They also compare favorably against their competitors across multiple synthetic data scenarios.

**Conclusion:**

The proposed HDMR-based approach appears to produce a reliable classifier that additionally allows one to describe how individual genes or gene-gene interactions affect classification decisions. Both real and synthetic data analyses suggest that our methods can be used to identify gene networks with dysregulated pairwise interactions, and are therefore appropriate for differential networks analysis.

## Background

The notion of a simple binary classification, as one of the corner stones of modern data analysis, has been considered in many different contexts and an abundance of algorithms have been proposed for this task. While research has recently shifted focus to classification rules in the context of big data, many bioinformatics applications deal with small-sample, high-dimensional prediction problems. Current high-throughput “omics” technologies measure tens of thousands of molecular features for each experimental unit (for instance, a patient’s tissue sample); however, research data is still usually limited to small sizes, rarely more than a few hundred units, impeding reliable analysis. Additionally, data might be heavily imbalanced, which adds to the challenge of correct classification in a small-sample, high-dimensional setting, with the minimum misclassification error criteria being too unreliable for consistent feature selection across multiple datasets [1, 2].

In contrast to many applications where machine learning methods are used merely to predict and do not have to provide explicit decision rules, the bioinformatics applications demand highly interpretable glass-box models to explain how a specific decision is obtained. In many instances it is important to know which features, e.g., genes, are used by the classifier, whether these features are biologically relevant, whether the distributional differences in features across two classes indicate biological variability or are merely artifacts of the measurement/normalization process, and what is the uncertainty of prediction at a new test point?

Answers to these questions are necessary to hypothesize about biological mechanisms of complex diseases such as cancer and to evaluate clinical utility of developed decision rules for tasks such as diagnosis and prognosis. But they are also necessary to explain how certain patterns in the data might motivate different actions, such as choosing a specific treatment over another for targeted therapy, exploring alternative treatments, or how to form hypotheses on the biological mechanisms that can potentially be targeted in drug discovery applications.

The small-sample high-dimensional nature of the problem, interpretability of outputted statistics, and complex feature dependencies, force the development of methods with few degrees of freedom that place strong assumptions (e.g. distributional assumptions) on the classification problem. For example, linear discriminant analysis (LDA) assumes features are Gaussian and have the same covariance matrix in both classes, quadratic discriminant analysis (QDA) assumes features are jointly Gaussian with different class-conditioned covariances, and a logistic regression model assumes that the log-likelihood ratio is an additive function of features. The common idea behind these methods is that although the “optimal” decision rule might be very complex (e.g., a high dimensional separating surface), it can be well approximated by a low dimensional model, and an appropriate family of models should contain a point close to the “best” low dimensional representation that can be reliably approximated given the observed data. In the current paper we follow a rather similar general approach, but apply a much more flexible method for deriving classifiers that allow for more flexible classification rules.

Recent studies emphasize the importance of gene synergies and genetic interactions for reliable analysis [3]. However, two general themes of the recent method developments are leveraging big data, such as the cancer genome atlas (TCGA), or taking advantage of side information such as sets of co-mutated genes or disease protein sub-networks, e.g. [4, 5]. Such information may not be readily available or may not be easily applicable to the current dataset, as cancer gene interactions are highly context dependent [6].

Utilizing pairwise interactions for reliable prediction aside, detecting disease-associated genetic interactions has been studied as a “gene discovery problem”. To that end, mutual information based synergy scores are proposed, e.g., [7, 8]. However, reliably inferring mutual information from data is a challenging task, which can be circumvented by quantizing expression values, building dendrograms based on expressions, utilizing ranked expressions instead of raw continuous values, or defining new statistics based on gene-pair expression rankings [7–10]. In [9] it is stated that dendrogram and doublet (a specific collection of transformations merging gene pair expressions into one-dimensional values) based methods are “helpless for discovering pair-wise gene interactions”. The information theoretic score of [8] cannot be easily utilized to test significance, using limited permutations of data to approximate the null, hypothesis which is computationally intensive [9]. Finally, [9] proposes a new conversion transformation, the absolute difference of ranked expressions constructing a t-statistic, which seems to balance performance and computation cost.

### High dimensional model representation

Consider a set of predictors as a random vector and a dependent variable as a function of predictors, e.g., class labels as a function of observed expressions. High dimensional model representation (HDMR) is a recently proposed framework to decompose functions of a random vector, i.e., the dependent variable, into a hierarchy of low dimensional models based on partial marginals of the full joint distribution [11]. Intuitively speaking, **HDMR expansion optimally decomposes a high dimensional non-linear system into a hierarchy of lower dimensional non-linear systems**, simplifying the process of studying each high-dimensional component. It enjoys several interesting properties. For example, the d^th^ order expansion is the best representation, in mean square error (MSE) sense, to estimate the dependent variable given its marginal distribution with all subsets of the predictors with at most *d* elements. Additionally, higher order expansion terms are independent of the lower order terms. HDMR assumptions are mild, only requiring certain moments to exist. Unfortunately, computing the HDMR expansion requires complete knowledge of the full joint distribution, and potentially solving large families of highly complex integral equations unless simplifying assumptions are made or special cases are considered [12]. This can be a deal breaker in many practical applications, where it is not always possible to obtain the full joint distribution given the small sample size. In this work, as a workaround, we propose algorithms that aim to approximate the HDMR expansion without directly estimating the full joint distribution and solving integral equations.

### Our contribution

The novelty of the proposed classification framework is three-fold. (1) Our approach provides a hierarchy of low dimensional representations of data, possibly allowing for analyzing progressively more complex interactions among features. (2) We propose a regression based approach to circumvent solving complex integral equations. (3) We can easily study the effects of any specified subsets of variables, and assess how their interactions affect the classifier output. As a side note, the proposed framework can also easily combine different parametric and non-parametric methods for computing log-likelihood ratios, an interesting property that adds further flexibility. However, we leave this extension for future work.

The paper is organized as follows. We first briefly overview the theory of HDMR expansion, and how it can be used for binary classification, considering in particular the special case of second order HDMR expansion. We then explain the regression-based algorithm of approximating the second order HDMR expansion and perform synthetic simulations comparing our method with several other popular classification rules proposed in the literature. Finally, we provide several real data examples, studying breast cancer, leukemia, and lung cancer.

## Methods

Here we describe our classification methodology based on the HDMR expansion, studied in detail in [11, 13]. We briefly review the theory, and then show how it applies to binary classification.

### HDMR expansion

HDMR provides a hierarchy of functions that describe how the interactions of variables affect the output. In particular, assuming output *Z* as a function of input random vector X=[X_*1*_,⋯,X_D_], i.e., Z=h(X), HDMR studies how h(X) can be decomposed to a hierarchy of partial observations. Let F={*1*,⋯,D}. The HDMR expansion of order *d* is the collection of functions h_u_(X_u_) for all u⊆F with |u|≤d that minimize the mean square error (MSE) of estimating *Z* given E(Z|X_u_) for all *u* under the condition that for all f∈u, E(h_u_(X_u_)|X_u∖f_)=*0*, which is equivalent to a hierarchical orthogonality criterion [13], i.e., HDMR terms of different orders are independent of each other. From [13] we have

1 $$\begin{array}{*{20}l}  h(X)&=h_{0}+\sum_{\substack{u \subseteq F \\ u \neq \phi}} h_{u}(X_{u}), \end{array} $$

2 $$\begin{array}{*{20}l}  h_{0}&=\int h(x) w(x) dx, \end{array} $$

3 $$\begin{array}{*{20}l}  h_{u}&=\int h(x) w(x_{-u}) dx_{-u}  \\ &- \sum_{v \subset u} h_{v}(x_{v}) - \sum_{v\neq u: v \cap u \neq \phi} \int h_{v}(x_{v}) w_{-u} dx_{-u}. \end{array} $$

Eq. 3 suggests that in the general case of dependent variables a component function, h_u_(x_u_), depends on all other expansion terms that have a non-empty intersection with *u*. However, assuming elements of *X* are independent, the last term of (3) equals zero. While this greatly simplifies the process of computing the HDMR expansion, the independence assumption can be heavily violated for expression data. We hereafter use E_d_(Z|X) to denote the d^th^ order HDMR expansion.

### Approximate second order HDMR for classification

We now focus on the second order HDMR expansion for correlated features. Observe that under the independence assumption, we have

4 $$\begin{array}{*{20}l}  E_{2}\left(Z|X\right)=w_{0}+w_{f} E\left(Z|X_{f}\right)+w_{f,f'} E\left(Z|X_{f,f'}\right), \end{array} $$

for some $w_{0},w_{f}, w_{f,f'} \in \mathbb {R}$. In case of dependent features, we still assume the second order HDMR expansion follows a structure similar to (4), except that the coefficients $\phantom {\dot {i}\!}w_{f}, w_{f,f'}$ are different than the independent case. Now, consider a binary classification problem with class labels y=*0*,*1* and feature index set *F*. Let *X* be a random unlabeled observation with true label y_x_. Given a training sample $\mathscr {S}$, it is desired to design a decision rule that assigns a label, $\hat {y}_{x}$ to *X* so that $\hat {y}_{x}=y_{x}$ with high probability. Note that given the full joint distribution of *X* and y_x_, one could have easily computed P(y_x_=*1*|X), or equivalently the log-likelihood ratio L(X)= log(P(y_x_=*1*|X)/P(y_x_=*0*|X)), and use a decision rule $\hat {y}_{x}=1_{L(X)>T}$, where *1*_q_ is the indicator function of statement *q* being correct and *T* is a threshold.

However, the full joint distribution is typically not available, and is usually estimated using $\mathscr {S}$. Alternatively, many models assume the classification rule belongs to a family parametrized by *θ*, which is estimated from $\mathscr {S}$. For example, a generalized linear model (GLM) with the logit link assumes $L(X)=\beta _{0}+ \sum _{f \in F}\beta _{f} X_{f}$, where X_f_ is the value of *X* for feature *f*. Here *θ* is the collection of *β*_*0*_ and *β*^f^’s. However, such model may be insufficient when pairwise feature interactions are of interest, and it can be difficult to train a GLM considering all potential pairwise interactions using LASSO and elastic net penalties due to the potentially large number of feature pairs. Assuming Z=L(X), and $\mathscr {S}$ is the training sample, we have

$$\begin{array}{*{20}l} E_{2}(L(X)|X,\mathscr{S})=c_{0}+\sum_{f \in F} c_{f} S(X_{f})+\sum_{f_{i},f_{j} \in F} c_{f_{i} f_{j}} S(X_{f_{i},f_{j}}),  \end{array} $$

for some $c_{0},c_{f},c_{f_{i},f_{j}} \in \mathbb {R}$ where

5 $$\begin{array}{*{20}l}  S(X_{f})&=E(\log L(X)|X_{f},\mathscr{S}), \end{array} $$

6 $$\begin{array}{*{20}l}  S(X_{f_{i},f_{j}})&=E(\log L(X)|X_{f_{i}f_{j}},\mathscr{S})-E(\log L(X)|X_{f_{i}},\mathscr{S})  \\ &-E(\log L(X)|X_{f_{j}},\mathscr{S}), \end{array} $$

It now remains to estimate coefficients c_f_ and $c_{f_{i},f_{j}}$, which is part of our classification algorithm discussed in the next section. Note that we also assume an external mechanism which already outputs $E(L(X)|X_{f},\mathscr {S})$ and $E(L(X)|X_{f_{i},f_{j}},\mathscr {S})$.

### Identifying pairwise feature interactions

An important application is identifying feature interactions that are significantly different between the classes, i.e., identify u={f_i_,f_j_}’s for which h_u_≢*0*. We have the following hypothesis test:

7 $$\begin{array}{*{20}l}  H_{0}: h_{u} \equiv 0 \ v.s. \ H_{1}: h_{u} \not \equiv 0. \end{array} $$

**Fig. 1:**
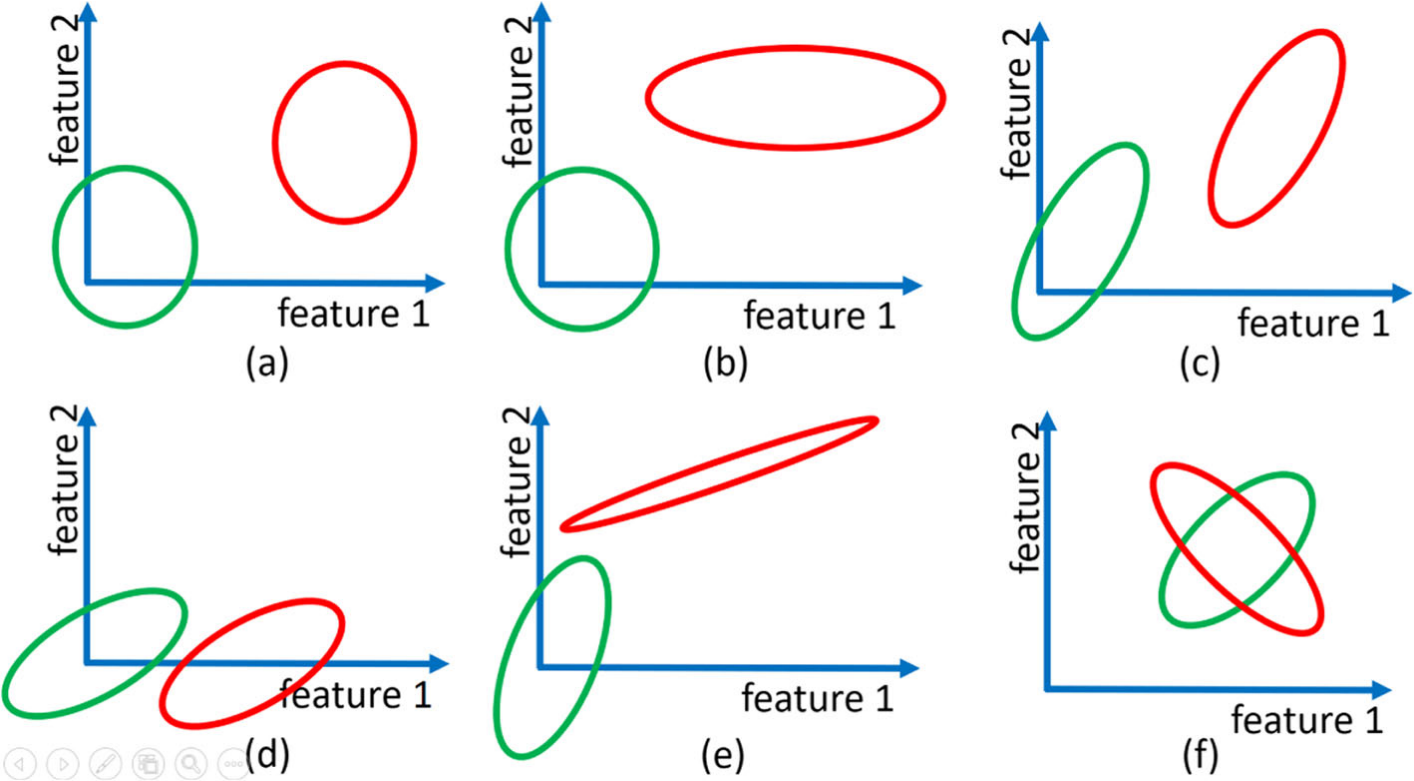
An example of different cases that might occur for two Gaussian features in a binary classification problem, where the two classes are denoted by red and green: (**a**) independent and equal variances, (**b**) independent and unequal variances, (**c**) correlated and equal covariances with degenerate means, (**d**) correlated and equal covariances with generic means, (**e**) correlated, unequal covariances, and unequal mean vectors, and (**f**) correlated, unequal covariances, and equal means vectors

Note this is a very difficult problem in general, and only the special case of Gaussian features is studied here. Assuming f_i_ and f_j_ are jointly Gaussian in each class, for an HDMR expansion of first order to be able to grasp the exact form of the log-likelihood ratio we need (1) all features of *u* to be independent given class label *y*, or (2) have the same covariance in both classes (assuming $\mu ^{f}_{0} \neq \mu ^{f}_{1}$ for all f∈F). In either case we have $L(X)=a_{0}+\sum _{f} a_{f} L(X|X_{f})$, for some coefficients $a_{0},a_{f} \in \mathbb {R}$. It is straightforward but tedious to show that other cases result in a second order expansion. Therefore, we can reformulate the hypothesis test of Eq. 7 as

8 $$\begin{array}{*{20}l}  &H_{0}: \rho^{f_{i},f_{j}}_{0}=\rho^{f_{i},f_{j}}_{1}=0 \ \text{ or} \Sigma^{f_{i},f_{j}}_{0} = \Sigma^{f_{i},f_{j}}_{1} \ \ \ \ v.s.  \\ &H_{1}: (\rho^{f_{i},f_{j}}_{0} \neq 0 \text{ or }\rho^{f_{i},f_{j}}_{1} \neq 0) \ \text{ and} \Sigma^{f_{i},f_{j}}_{0} \neq \Sigma^{f_{i},f_{j}}_{1}, \end{array} $$

where $\rho ^{f_{i},f_{j}}_{y}$ and $\Sigma ^{f_{i},f_{j}}_{y}$ are the correlation coefficient and covariance matrix of feature pair f_i_,f_j_ in class *y*, respectively. Figure 1 provides several examples on cases with and without pairwise feature interactions. In cases (a), (b), and (d) there are no pairwise feature interactions. Case (c) denotes a degenerate case and is studied in the Supplementary. Cases (e) and (f) depict feature pairs with pairwise interactions.

Testing conditional independence is a statistically difficult problem [14], and has been studied for certain cases in [14]. As an approximation, we adopt the following approach. Let $P^{f_{i},f_{j}}_{y}$ be the *p*-value of an independence test performed on data in class *y*. We use the Pearson linear correlation test for Gaussian data. Assuming points in different classes are independent, we treat them as independent tests, and use Fisher’s method for meta-analysis: $C=-2 \left (\log \left (P^{f_{i},f_{j}}_{0}\right)+ \log \left (P^{f_{i},f_{j}}_{1}\right)\right)$ follows a *χ*^*2*^ distribution with 4 degrees of freedom under the null, giving us the final *p*-value. We use the likelihood ratio test of [15] with the *χ*^*2*^ adjustment to find *p*-values of *Σ0*f_i_,f_j_=*Σ1*f_i_,f_j_. Finally, we use the union bound and add the two *p*-values to obtain our final *p*-value, which is an overestimate. We hereafter call this approach *multiple test mixing for pairwise interactions* (MTM) and note that it is appropriate for differential network analysis. Indeed, identifying feature pairs with interesting interactions, i.e., pairwise dependencies that require looking at a second order expansion, instead of a first order expansion, are a goal of co-expression and differential network analysis. Different modes of co-expression is discussed in [16], and some of its applications, such as expression analysis, functional classification, and gene-disease prediction, are described in [17, 18]. Differential network analysis is further discussed in [19, 20].

## The classification algorithm

Here we describe our approach to build a classifier that is inspired by second order HDMR expansion of the log-likelihood ratio. Again suppose sample $\mathscr {S}$ is collected, and for each set *u* such that |u|≤*2*, we have a method that outputs the log-likelihood ratio of the test point belonging to class 1, i.e., we have a method that outputs $L(X_{u}|\mathscr {S})$ for all *u* such that |u|≤*2*. Given all these values, it remains to combine them into a test score. However, in a small-sample problem the number of feature pairs can be much larger than sample size. This creates an ill-posed problem as the number of equations is smaller than the number of parameters to estimate. Therefore, many classical methods for obtaining the model parameters from data, such as maximum likelihood, are not applicable. Not being able to compute the exact second order HDMR expansion by framing it as a regression problem is the main reason we need to look at alternative training methods, and is the main reason we label the concomitant classifier design approach as “HDMR expansion inspired” or as an “HDMR expansion perspective” for classification.

To circumvent the ill-posed problem, we use a variation of objective functions mostly studied in compressed sensing, e.g. [21], that estimate a sparse signal given 1-bit quantized observations. The connection between these objectives and a convex relaxation to the logistic regression problem is discussed in [22]. Heuristically speaking, given a feature vector in the form of log-likelihood ratios of partial observations x_u_, here we find weights that maximize the distance between the average points of each class. The heuristic for using such objective function is as follows. On one hand, HDMR obtains the weights that result in the “best” low dimensional representation, i.e., the MSE estimate of the log-likelihood ratio, yielding low prediction errors. On the other hand, weights that maximize the distance between the projections of the center points of the two classes into a one dimensional space should also yield low prediction error. Hence, such objective function should result in a model with an error probability close to that of the HDMR expansion. Here we have used the HDMR theory to obtain the functional form of the solution, and use algorithms borrowed from 1-bit compressed sensing to estimate the HDMR coefficients.

**Fig. 2:**
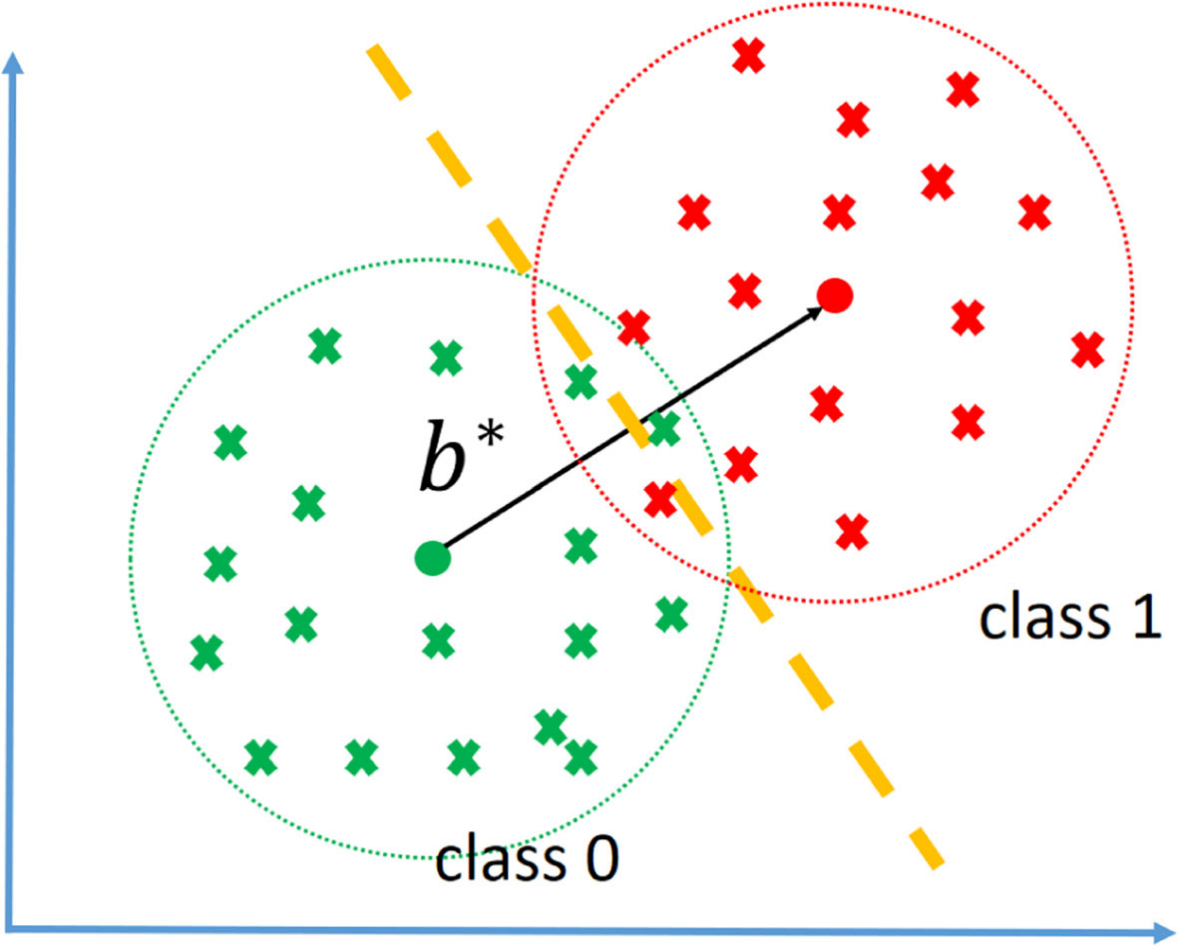
The illustration of the b^∗^ selection process. Given V(X) vectors for the two classes, denoted by red and green crosses, the b^∗^ is chosen to maximize the distance between the center of projections of V(X) vectors on *b*

**Fig. 3:**
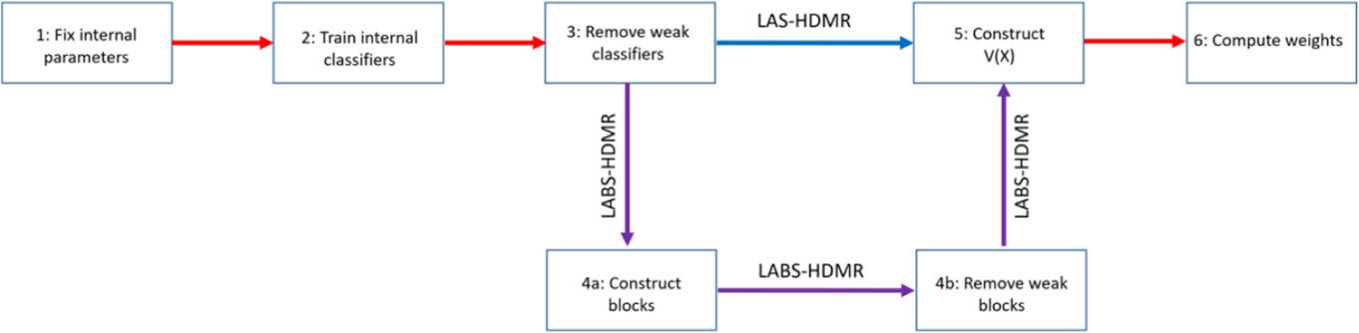
The overall pipeline of LAS-HDMR and LABS-HDMR. In step 1 the algorithm parameters and the internal classification machineries are selected. In step 2 classifiers using individual features and feature pairs are trained and log likelihood ratios are saved. In step 3 weak classifiers (based on r^f^ and $|{r}^{{f}_{i},{f}_{j}}|$) are removed. LAS-HDMR moves to step 5, but LABS-HDMR moves to steps 4a and 4b, in which feature pairs construct blocks, and weak blocks are removed. In step 5 the final feature vector V(X) is constructed and in step 6 the weights used to combine elements of V(X) are computed

In many high-dimensional statistics applications, it is common to favor some bias to reduce estimation variance. Examples and a detailed discussion on this issue is provided in [23]. In order to reduce variance in our setting, we remove the weakest classifiers, in forms of single feature classification rules or relatively independent feature pairs whose information is already provided in the first order expansion. We compute

9 $$\begin{array}{*{20}l}  r^{f}=\frac{1}{n_{1}} \sum_{x \in \mathscr{S}_{1}} L(x_{f}|\mathscr{S}) - \frac{1}{n_{0}} \sum_{x \in \mathscr{S}_{0}} L(x_{f}|\mathscr{S}), \end{array} $$

where $\mathscr {S}_{y}$ is the restriction of $\mathscr {S}$ to points in class *y*, and n_y_ is sample size in class *y*. We remove features for which r^f^<T_*1*_, where T_*1*_ is a threshold. For feature pairs we compute $\phantom {\dot {i}\!}r^{f_{i},f_{j}}$ defined as

10 $$\begin{array}{*{20}l}  \frac{1}{n_{1}} \sum_{x \in \mathscr{S}_{1}} L(x_{f_{i},f_{j}}|\mathscr{S}) - \sum_{x \in \mathscr{S}_{0}} \frac{1}{n_{0}} L(x_{f_{i},f_{j}}|\mathscr{S})-r^{f_{i}}-r^{f_{j}}, \end{array} $$

and remove feature pairs for which $\phantom {\dot {i}\!}|r^{f_{i},f_{j}}|<T_{2}$, for some threshold T_*2*_. Feature pairs for which $r^{f_{i},f_{j}}>T_{2}\phantom {\dot {i}\!}$ are risk increasing pairs, and feature pairs for which $r^{f_{i},f_{j}}<-T_{2}\phantom {\dot {i}\!}$ are risk decreasing pairs. We now find weights that combine $S(X|X_{u},\mathscr {S})$ of feature and feature pairs with large r^f^ and $|r^{f_{i},f_{j}}|\phantom {\dot {i}\!}$, respectively. Although the HDMR expansion of the log-likelihood ratio is unique, the actual true weights may be complicated to derive. Since we mostly compare the HDMR expansion against a specific threshold to assign a label to a newly observed point, we may consider the following optimization problem to approximately solve for the desired weights.

11 $$\begin{array}{*{20}l}  b^{*}={\text{argmax}}_{b:||b||_{2}=1} \left(\frac{1}{n_{1}} \sum_{x \in \mathscr{S}_{1}} b.V(x) -\frac{1}{n_{0}} \sum_{x \in \mathscr{S}_{0}} b.V(x)\right), \end{array} $$

where V(x) is the collection $S(X_{f}|\mathscr {S})$’s for all features *f* such that r^f^>T_*1*_ and $S(X_{f_{i},f_{j}}|\mathscr {S})$ of all feature pairs f_i_,f_j_ such that $\phantom {\dot {i}\!}|r^{f_{i},f_{j}}|>T_{2}$, and “.” denotes inner product. Figure 2 depicts how b^∗^ is selected given vectors V(X) for the training data. Given a new observation *X*, we find R(X)=b^∗^.V(X), and we assign class label $\hat {Y}=1_{R(X)>T}$, where *T* is a threshold. Note T_*1*_, T_*2*_, and *T* are parameters of the model. Here they are selected using a grid search within cross validation; however, efficient parameter tuning strategies should be explored (see the Conclusion and Future Work section). We hereafter refer to this classification rule as *linear approximation second order HDMR expansion* (LAS-HDMR). The pseudo-code of LAS-HDMR is provided in Algorithm 1. To summarize, given the data, the machinery that outputs log likelihood ratios of features and feature pairs, and the algorithm parameters, LAS-HDMR computes the risks of individual features and feature pairs, removes weak ones, and computes the weights that maximize the distance between the centers of each class. The overall pipeline is provided in Fig. 3. 
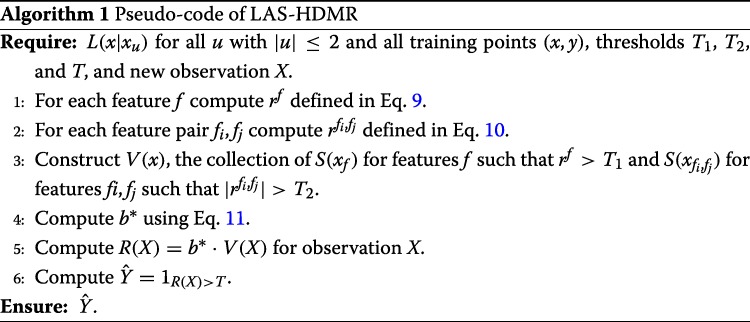

### The block model extension

The optimization problem in Eq. 11 maximizes the Euclidean distance between the centers of points in different classes, which might be most suitable for cases where elements of V(x) are independent. However, the feature pairs in LAS-HDMR can be heavily correlated. Therefore, it might be useful to merge heavily correlated feature pairs in blocks, average feature pairs of each block, and use the average log-likelihood of each block in V(x). Note that almost any community detection algorithm over graphs can be used to cluster feature pairs into blocks, where each node is a feature pair and the edges measures the correlations between log-likelihood ratios of the two feature pairs (see [24, 25] for a review on graph community detection). We consider the following simple block construction scheme. For each feature f_i_ construct risk increasing and risk decreasing blocks $\phantom {\dot {i}\!}P^{f_{i}}$ and $\phantom {\dot {i}\!}N^{f_{i}}$, respectively, as follows:

12 $$\begin{array}{*{20}l} P^{f_{i}}&=\{f_{j}: f_{j} \neq f_{i}, r^{f_{i} f_{j}}>T_{2} \}, \end{array} $$

13 $$\begin{array}{*{20}l} N^{f_{i}}&=\{f_{j}: f_{j} \neq f_{i}, r^{f_{i} f_{j}}<-T_{2} \}. \end{array} $$

Afterwards, for each block $\phantom {\dot {i}\!}P^{f_{i}}$ and $N^{f_{i}}\phantom {\dot {i}\!}$ compute $r^{P^{f_{i}}}\phantom {\dot {i}\!}$ and $r^{N^{f_{i}}}\phantom {\dot {i}\!}$, the risks of positive and negative risk feature pairs containing f_i_, being the average risks of feature pairs in the block. We then remove “weak” blocks. Weak risk increasing blocks are those for which $r^{P^{f_{i}}}<T_{3}\phantom {\dot {i}\!}$, and weak risk decreasing blocks are those for which $r^{N^{f_{i}}}>-T_{3}\phantom {\dot {i}\!}$. Note T_*3*_ is a parameter of the model that is tuned via a grid search within cross validation. Now, given observation *x*, V(x) is comprised of the log-likelihood ratio of single features for which r^f^>T_*1*_ and average $\phantom {\dot {i}\!}S(X_{f_{i},f_{j}})$ of risk increasing and risk decreasing blocks that had absolute average risk large than T_*3*_. We again use Eq. 11 to obtain HDMR coefficients. Finally, given new observation *X*, V(X) is formed, R(X)=b^∗^.V(X) is computed, and $\hat {Y}=1_{\{R(X)>T\}}$ is the predicted label. We hereafter call this classification algorithm *linear approximation of block second order HDMR expansion* (LABS-HDMR). The pseudo-code of LABS-HDMR is described in Algorithm 2, and the overall pipeline is provided in Fig. 3. 
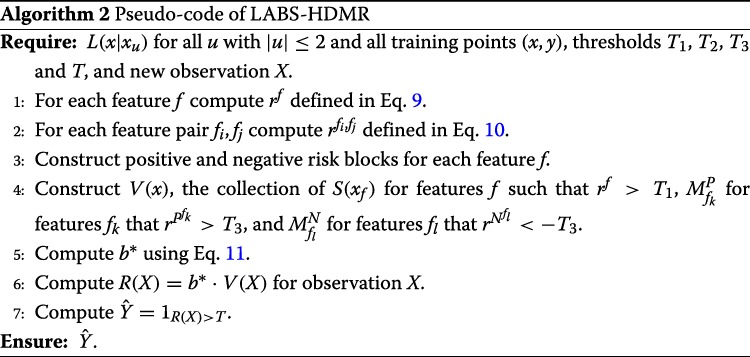


## Synthetic simulations

**Fig. 4:**
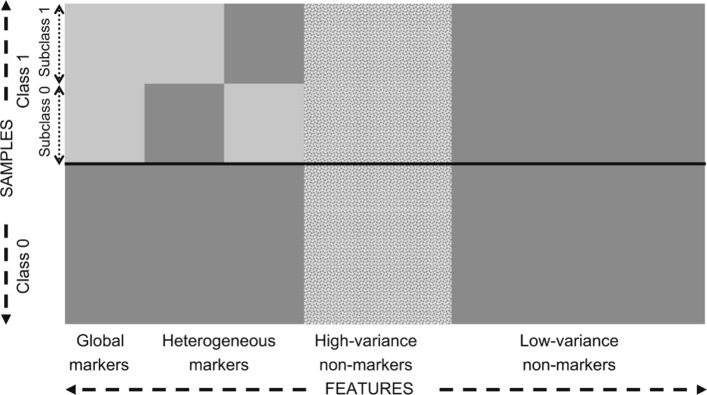
An illustration of global markers, heterogeneous markers, and non-markers of the data generation model for continuous observations, adopted from [26]

Here we perform several simulations to study LAS-HDMR and LABS-HDMR classifiers in more detail. We use a synthetic model developed to mimic microarrays and gene expression levels for data generation. The original model is proposed in [26], and has been extended in [27, 28]. Here we use the extended model of [27] in which features are markers or non-markers. Markers are either global or heterogeneous, and comprise blocks of size *k*, where features in the same block are dependent and features in different blocks are independent of each other. Each block of global markers in class 0 is Gaussian with zero mean and covariance $\sigma ^{2}_{0} \Sigma _{0}$, where diagonal elements of *Σ*_*0*_ are 1 and off-diagonal elements are *ρ*_*0*_. Global markers in class 1 are either synergetic or marginal. In the synergetic case mean vector of each block in class 1 is [*1*,*1*/*2*,⋯,*1*/k], and in the marginal case it is [*1*,*0*,⋯,*0*]. The covariance matrix of the Gaussian distribution is $\sigma ^{2}_{1} \Sigma _{1}$. Diagonal elements of *Σ*_*1*_ are 1 and off diagonal elements are *ρ*_*1*_. Heterogeneous markers are similar to global markers in class 0 but comprise *c* subclasses in class 1, where in each subclass certain points follow a distribution similar to class 1 global markers and the remaining points are similar to class 0 markers. Non-markers are either low variance or high variance. Low variance non-markers are similar to class 0 markers. High variance non-markers are independent of each other, and each HV non-marker follows a Gaussian mixture of the form $pN(0,\sigma ^{2}_{0})+(1-p)N(1,\sigma ^{2}_{1})$, where *p* is drawn uniformly at random over the interval [*0*, *1*]. Note $\sigma ^{2}_{0}$, $\sigma ^{2}_{1}$, *ρ*_*0*_, *ρ*_*1*_, *k*, and *c* are parameters of the model. Figure 4, adopted from [26], shows an illustration of the feature types developed in this synthetic model.

We now study how LAS-HDMR and LABS-HDMR perform under this model. In this simulation, all features are heterogeneous markers, to create a more difficult problem. We fix |F|=*60*, c=*2*, k=*10*, $\sigma ^{2}_{0}=0.25$, and $\sigma ^{2}_{1}=0.64$ and consider 4 scenarios: (1) synergetic markers with *ρ*_*0*_=*ρ*_*1*_=*0*.*5*, (2) synergetic markers *ρ*_*0*_=*0*.*1*, and *ρ*_*1*_=*0*.*9*, (3) marginal markers with *ρ*_*0*_=*ρ*_*1*_=*0*.*5*, and (4) marginal markers with *ρ*_*0*_=*0*.*1* and *ρ*_*1*_=*0*.*9*. We generate a stratified sample of size *n* (to be specified below) with an equal number of points in each class for training, and a stratified sample of size 2000 with an equal number of points in each class for testing. Given training data, several classifiers are trained, which are then applied to the test data. We compute the receiver operator characteristic (ROC) curve and the area under curve (AUC) averaging over 100 iterations. Note large test sets were used to accurately compute prediction errors. Despite the test data being balanced, we believe AUC is a more reliable performance statistic than accuracy, as experimental data are typically imbalanced. That being said, in the current setup, for each point on the ROC curve obtained for threshold *T*, accuracy is *1*−*0*.*5*(P_I_+P_*II*_), where P_I_ and P_*II*_ are probabilities of Type I and Type II errors, respectively.

In addition to LAS-HDMR and LABS-HDMR we implement the following classifiers for comparison: regularized quadratic discriminant analysis (RQDA) with regularization value ranging from 0 to 1 in steps of 0.1, regularized linear discriminant analysis (RLDA) with regularization value ranging from 0 to 1 in steps of 0.1, linear support vector machine (SVM), random forest (RF) with the number of tree ranging from 10 to 100 in steps of 20, *k* nearest neighbors (kNN) with k=*3*,*5*,⋯,*30*, and generalized linear models with linear and quadratic probit links using LASSO and elastic net penalties (*α*=*0*.*5*) with penalty coefficients *λ*=*0*.*01*:*0*.*01*:*0*.*1*. These methods are discussed in detail in [29–31].

**Fig. 5:**
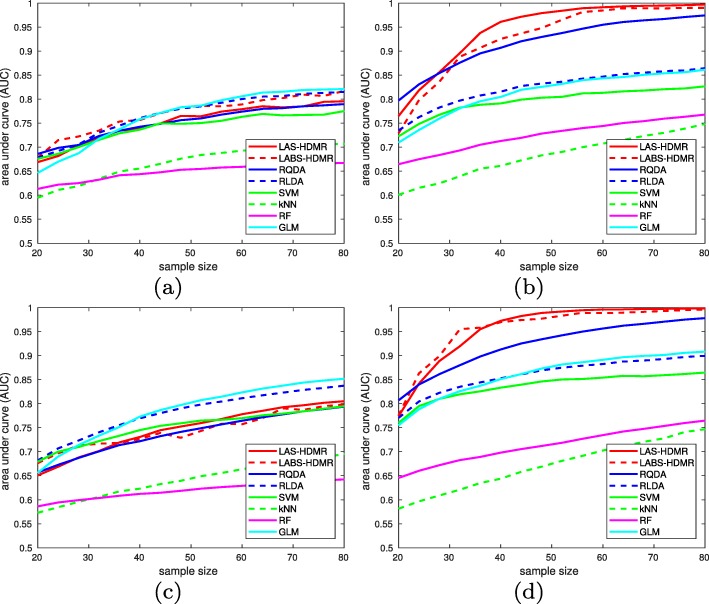
AUC of different classification algorithms versus sample size for different data generation models: (**a**) synergetic features and equal correlations, (**b**) synergetic features and unequal correlations, (**c**) marginal features and equal correlations, and (**d**) marginal features and unequal correlations

For each family with multiple tuning parameters and for each sample size, we report the largest AUC among all tested parameter values over the test data. Figure 5 plots the AUCs over test data averaging over 100 iterations as the sample size increases from 20 to 80 in steps of 4. When features have similar correlation matrices in both classes, classical methods such as RLDA and RQDA perform best and are closely followed by LAS-HDMR and LABS-HDMR. However, when correlation coefficients differ between the two classes LAS-HDMR and LABS-HDMR outperform other tested classifiers. Here we observe little difference between LAS-HDMR and LABS-HDMR, suggesting we do not need to merge feature pairs into blocks and the number of feature pairs to consider is not too large for this problem. We leave a more thorough comparison of LAS-HDMR and LABS-HDMR for future work. ROC plots are provided in the Supplementary.

Note that we started from the problem of finding the “best” second order representation of the log-likelihood ratio, but, due to computational difficulties, had to make several assumptions and approximations along the way. Therefore, it is possible that we end up mis-specifying the exact second order HDMR decomposition. In such scenarios, it can be very probable that another method outperforms LAS-HDMR and LABS-HDMR. Note LAS-HDMR and LABS-HDMR enjoy competitive overall performance in all tested scenarios, and outperform other methods when correlation coefficients differ between the classes. They are competitive methods, and hence can be suitable for a wide range of problems. Note that settings where LAS-HDMR and LABS-HDMR do not perform best correspond to those in which correlation coefficients are equal in both classes, for which RLDA and linear probit models perform best. This suggests maybe in these cases the first order HDMR expansion is more appropriate to represent the data (LDA is equivalent to a first order HDMR expansion under its modeling assumptions), although variances are slightly different between the classes. The small sample sizes used in this simulation may impede quadratic classifiers to satisfactorily estimate the distribution parameters, which may result in their poor performance. Similar patterns are observed in [32, 33]. Additionally, given that the first order expansion is sufficient to represent data, a second order HDMR model might suffer curse of dimensionality.

### Identifying pairwise interactions

**Fig. 6:**
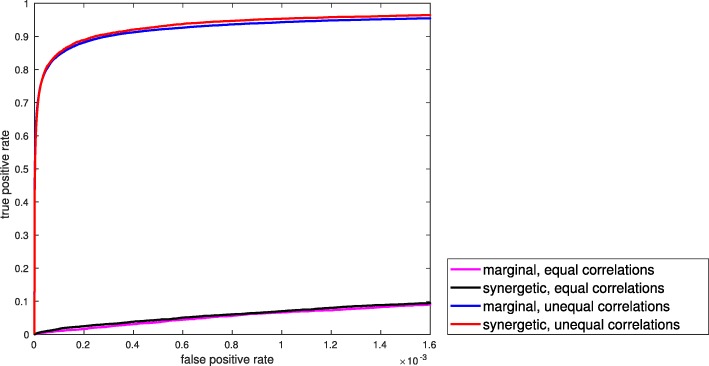
ROC curves of MTM method for detecting pairwise feature interactions for different marker and correlation types when n=*40*. Marker’s class-conditioned means have almost no effect on the ROC curves as we look for certain structures on the covariance matrix; however, correlation structures have a much larger impact on ROCs. Probability of detection of much lower when correlations are equal for a fixed false alarm rate

**Fig. 7:**
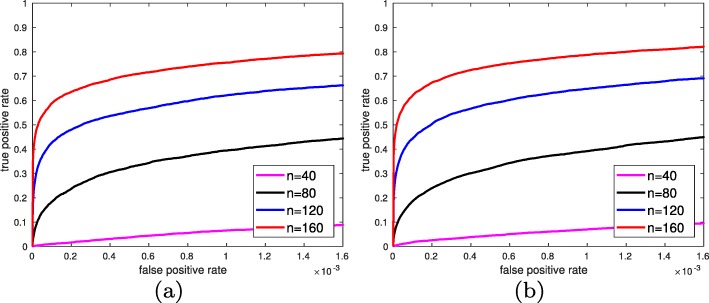
ROC curves of MTM for detecting pairwise interactions for different sample sizes for (**a**) marginal and (**b**) synergetic markers with equal correlations in both classes. Marker means have almost no effect on the ROCs. MTM’s ability to grasp dysregulated interactions in the challenging case of equal correlation coefficients rapidly improves as sample size increases

Here we evaluate the performance of MTM in identifying significant pairwise feature interactions. A comparison of MTM with the method of [9] is provided in the Supplementary. We fix |F|=*5000*, with 20 global markers, 80 heterogeneous markers, and 2000 high variance non-markers. We again assume k=*10*, c=*2*, $\sigma ^{2}_{0}=0.25$, $\sigma ^{2}_{1}=0.64$, and consider the 4 scenarios of the previous section for mean types and correlation coefficients. Figure 6 provides the ROC curves of MTM for different marker and correlation values when n=*40*, averaging over 100 iterations. MTM performs best when correlations are different between the two classes: red and blue lines denoting unequal correlations for synergetic and marginal markers, respectively, enjoy a higher probably of detection compared with black and magenta lines denoting equal correlations for synergetic and marginal markers, respectively. Note the mean types (marginal or synergetic) have little effect on the ROC curves. Figure 7 provides ROC curves of the equal correlation cases for different sample sizes, averaging over 100 iterations. As expected, with increase in the sample size it becomes easier to detect pairwise interactions. To benchmark MTM we compared it with the absolute conversion method of [9], which is proposed as a “fast” algorithm. However, we observed it is computationally more intensive than MTM. We considered marginal and synergetic markers with equal correlations, fixed n=*40*, and reduced the number of iterations to 50. Results are provided in the Supplementary, in which MTM outperforms the method of [9].

## Experimental data analysis

We apply LAS-HDMR, LABS-HDMR, and the comparison classifiers of the previous section to datasets studying relapsing breast and lung cancer patients. We also evaluate if MTM can detect significant pairwise gene interactions in realistic settings. We specifically selected datasets resulting in tasks more challenging than healthy versus normal labels. Such datasets are in particular challenging as data can be small in size and imbalanced (only a small portion of followed up patients may relapse). Additionally, breast and lung cancers are well studied in the literature, allowing us to evaluate if the detected patterns are biologically plausible. A leukemia dataset is studied in the Supplementary.

### Breast cancer

The data obtained in [34] and [35] deposited on gene expression omnibus (GEO) database [36] with accession number GSE25066, containing expression levels of 397 relapse free and 111 relapsing breast cancer patients, all of whom went through neoadjuvant taxane-anthracycline chemotherapy. Data is based on the GPL96 platform, and is already pre-processed and normalized. The dataset contains 22,283 probes, of which 20,967 map to genes. We only use probes that map to genes in our analysis. First, 100 relapsing and 360 non-relapsing patients randomly select as training data, and the remaining points are used for testing. The likelihood ratio test (LRT) statistic of [37], which is equivalent to the optimal Bayesian filter scoring function under independent Gaussian models [27, 38] under Jeffreys prior, is used to select the top 100 differentially expressed genes. We iterate 100 times.

**Fig. 8:**
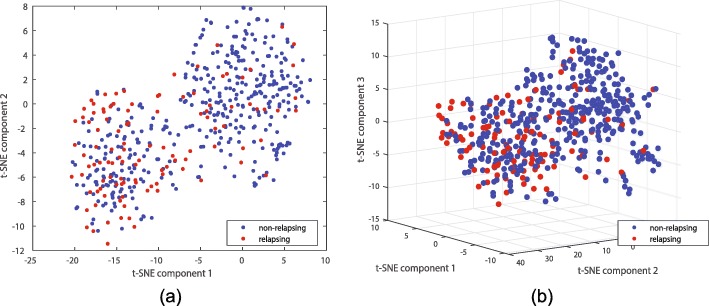
t-SNE plots of the breast cancer dataset separating the relapsing (high risk) and non-relapsing (low risk) patients: (**a**) 2D and (**b**) 3D plots. Blue and red denote the non-relapsing and relapsing patients, respectively. Few relapsing patients resemble non-relapsing patients while a larger portion of non-relapsing patients resemble relapsing patients, suggesting each class may be polluted with a sub-population of points that truly belong to the other class

**Fig. 9:**
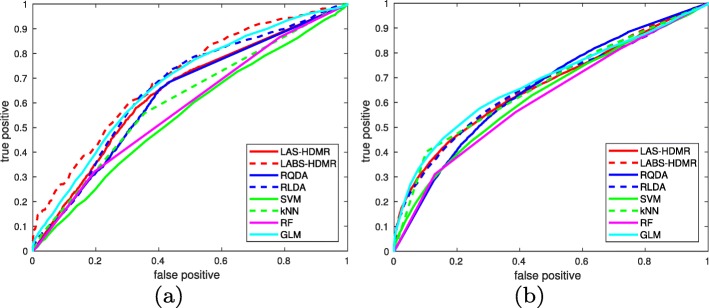
ROC curve of different classifiers for the (**a**) breast and (**b**) lung cancer datasets: (**a**) In the breast cancer dataset LABS-HDMR uses RQDA to infer log-likelihood ratio of partial observations, greatly improves RQDA’s accuracy, and is followed by GLMs. LAS-HDMR closely follows GLM and RLDA, uses RQDA in its design, improves RQDA’s accuracy for small false positives, and performs similar to RQDA for large false alarms. (**b**) In the lung cancer dataset GLM performs best, but is closely followed by LAS-HDMR and LABS-HDMR, which both improve on the AUC of the intrinsic machinery they use for computing log-likelihood ratio of partial observations

2D and 3D t-SNE [39] plots using the cityblock distance are provided in Fig. 8, suggesting the two classes do separate. It seems each class contains a few points which may truly belong to the other class, i.e., each class is polluted with a small subpopulation truly belonging to the other class. Alternatively, larger follow-up times may be necessary to further determine if certain non-relapsing patients relapse, and hence should belong to class 1. The large number of non-relapsing patients that resemble relapsing patients reduces the measured AUC. Table 1 lists the AUC of different classifiers on this dataset (over the hold out test data). Figure 9a provides the ROC plots.

**Table 1:** AUC of classification algorithms for the cancer datasets

method	LAS-HDMR	LABS-HDMR	RQDA	RLDA	SVM	RF	kNN	GLM
breast cancer	64.21%	**69.95%**	62.70%	66.04%	55.34%	60.87%	58.18%	67.55%
lung cancer	66.56%	67.67%	65.47%	66.62%	63.03%	66.87%	61.60%	**68.22%**

**Fig. 10:**
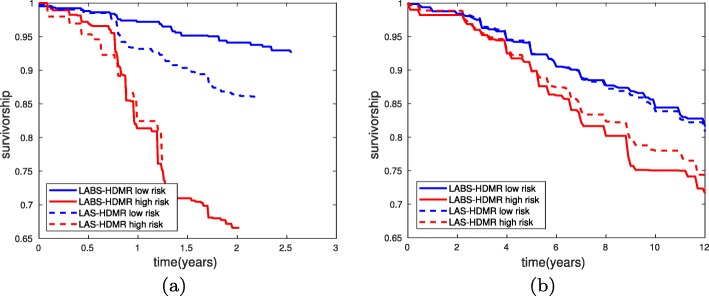
Kaplan-Meier survivorship plots for the (**a**) breast and (**b**) lung cancer datasets. (**a**) In the breast cancer dataset high/low risk patients have a high/low chance of relapse withing the follow-up time. (**b**) In the lung cancer dataset high/low risk patients have a high/low chance of relapse or death due to cancer withing the follow-up time. Both LABS-HDMR and LAS-HDMR do separate the patients given input expression levels

As Table 1 suggests, all methods do not enjoy a high AUC. We observed that the variant of LAS-HDMR using RQDA with *λ*=*0*.*8* achieved the highest AUC. The largest AUC for the variant of LAS-HDMR using RLDA was *63*.*12%* obtained for *λ*=*0*.*9*. In contrast, LABS-HDMR seems to enjoy the highest AUC, obtained using RQDA with *λ*=*0*.*1*, which is the closest tested variant to conventional QDA. This may suggest that a second order expansion is not satisfactory enough for this dataset, emphasizing the need to look at higher order expansions. Finally, Fig. 10a provides the Kaplan-Meier survivorship plots based on the assigned labels to the test data, averaging over 100 iterations for LAS-HDMR and LABS-HDMR. The figure provides extra assurance that indeed the proposed algorithms separate the two classes, the approximate second order HDMR expansion of the log-likelihood ratio, i.e., R(X), is an appropriate statistic to denote the “risk” of an event, and the proposed methods can be further used in conjunction with other data analysis tools. As t-SNE plots in Fig. 8 suggest, many low risk patients resemble high risk patients, and we expect a well-designed classification rule to mislabel such points; otherwise, the separating plane (curve) should be extremely complex, raising serious concerns of over-fitting. This explains why many high risk patients have not relapsed up to the follow-up time.

Although LAS-HDMR and LABS-HDMR may not yield high classification accuracy similar to other classification algorithms, their glass box nature simplifies the process of identifying genes and gene pairs that contribute the most to the classifier’s prediction. We use all of the data for training, and use RQDA with *λ*=*0*.*8* to obtain the log-likelihood ratios. Table 2 lists the top 10 genes of LAS-HDMR and their risks. Many of the top genes, such as IL8 [40], also known as C-X-C motif chemokine ligand 8 (CXCL8), and growth regulating estrogen receptor binding 1 (GREB1) [41] are suggested to be affected in breast cancer. Table 3 lists the top 10 LAS-HDMR gene pairs. Comparing Tables 2 and 3 we observe that gene interactions tend to have a larger risk than individual features. Note all risks describe the average increase/decrease of the log likelihood ratio, and are hence on the same scale for all genes and gene pairs. Scatter plots of several gene pairs with interesting interactions is provided in the Supplementary). For example, we observed either GREB1 or carboxypeptidase B1 (CPB1) are over-expressed among non-relapsing patients, and under-expression of both GREB1 and CPB1 is necessary to have a high risk of relapse. Finally, many of the top gene-gene interaction pairs contain GREB1, signal peptide CUB domain EGF-like 2 (SCUBE2), GATA Binding Protein 3 (GATA3), and IL8, suggesting their interaction might be key to studying breast cancer. The variant of LABS-HDMR that achieved the highest AUC used RQDA with *λ*=*0*.*1*, and is studied in detail in the Supplementary.

**Table 2:** Top breast cancer genes used for classification by LAS-HDMR

Rank	Gene	Risk	Rank	Gene	Risk
1	ORM1, ORM2	0.92	6	ACADSB	0.75
2	IL8	0.87	7	PTOV1	0.75
3	ZNF395	0.85	8	ZNF673	0.75
4	GREB1	0.8	9	AR	0.74
5	TBC1D9	0.78	10	LGALS8	0.71

**Table 3:** Top LAS-HDMR gene pairs of the breast cancer dataset

Rank	Gene 1	Gene 2	Risk
1	GREB1	SCUBE2	2.24
2	GREB1	CPB1	2.23
3	ORM1, ORM2	GREB1	2.22
4	GREB1	IL8	2.22
5	ZNF395	GREB1	2.1
6	GREB1	GATA3	2.07
7	GREB1	NAT1	2.06
8	GREB1	TBC1D9	2.05
9	GREB1	ACADSB	2.03
n10	ORM1, ORM2	SCUBE2	2.02

**Fig. 11:**
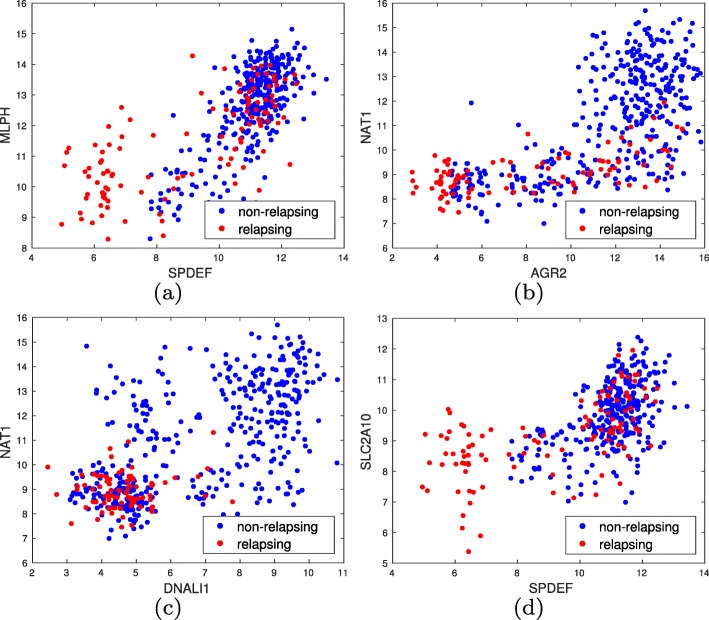
Scatter plots of gene pairs with significant interactions for the breast cancer dataset. Red denotes relapsing breast cancer patient within the follow-up time and blue denotes patients who did not relapse: (**a**) decreased expression of SPDEF and MLPH increases relapse risk, (**b**) over-expression of both AGR2 and NAT1 is an indicator of low relapse risk, decreased expression of NAT1 and over-expression of AGR2 seems to result in medium risk, and patients for whom both AGR2 and NAT1 have decreased expressions are in high risk of relapse, (**c**) to have a high risk of relapse both DNALI1 and NAT1 must have decreased expression, i.e., over-expression of either gene is an indicator of low relapse risk, (**d**) decreased expression of both SPDEF and SCL2A10 is an indicator of high relapse risk

Now we look for significant pairwise gene interactions. First, for each gene, we only consider the probe ranking highest by LRT so that probes mapping to the same genes do not disrupt the analysis, resulting in 13,211 different genes. This results in 87,258,655 tests. We observed that MTM can be heavily affected by small subpopulations, heavy tails, and outliers, and therefore used MATLAB’s built in isoutlier function with its default settings to remove potential outliers before further analysis. Bounding the false discovery rate (FDR) by *5%* using the Benjamini-Hochberg (BH) procedure [42] 1,275,351 pairwise interactions are significant (about *1*.*46%* of tested hypotheses). Table 4 lists several of the top gene pairs and their adjusted *p*-values. Figure 11 provides scatter plots of several gene pairs. We observe many interesting patterns that require further investigation: (1) under-expression of both SAM pointed domain containing ETS transcription factor (SPDEF) and MLPH, also known as synaptotagmin-like protein 2A (SLAC2A), increases the risk of relapse, (2) over-expression of anterior gradient protein 2 homolog (AGR2) and N-Acetyltransferase 1 (NAT1) is an indicator of low risk, over-expression of AGR2 and under-expression of NAT1 is an indicator of “medium” risk, and under-expression of both AGR2 and NAT1 is an indicator of high risk, (3) over-expression of NAT1 or DNALI1 is an indicator of low relapse risk. Comparing Fig. 11a and d suggests solute carrier family 2 member 10 (SLC2A10) and MLPH are heavily correlated with a positive correlation coefficient, which is indeed observed in the data as well.

**Table 4:** Top breast cancer gene pairs and adjusted *p*-values (×*10*^−*25*^)

Rank	gene 1	gene 2	adj *p*-value
1	SPDEF	MLPH	<*10*^−*4*^
2	MSN	SPDEF	<*10*^−*4*^
3	SCGB2A2	SCGB1D2	<*10*^−*4*^
4	TFF3	SPDEF	<*10*^−*4*^
5	RHOB	SPDEF	<*10*^−*4*^
11	SLC44A4	SPDEF	0.0014
12	SPDEF	FAM174B	0.0064
13	FOXA1	NAT1	0.0128
14	GATA3	SPDEF	0.0477
15	TSPAN1	SPDEF	0.0477

**Fig. 12:**
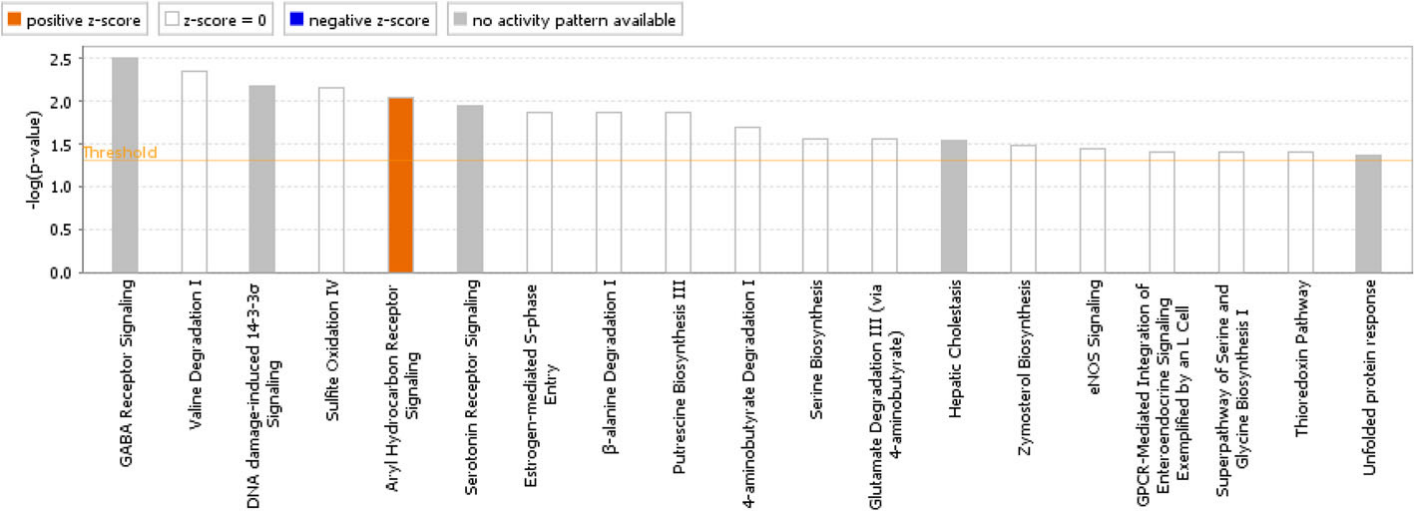
Top IPA canonical pathways of the largest MTM gene cluster of the breast cancer dataset

**Fig. 13:**
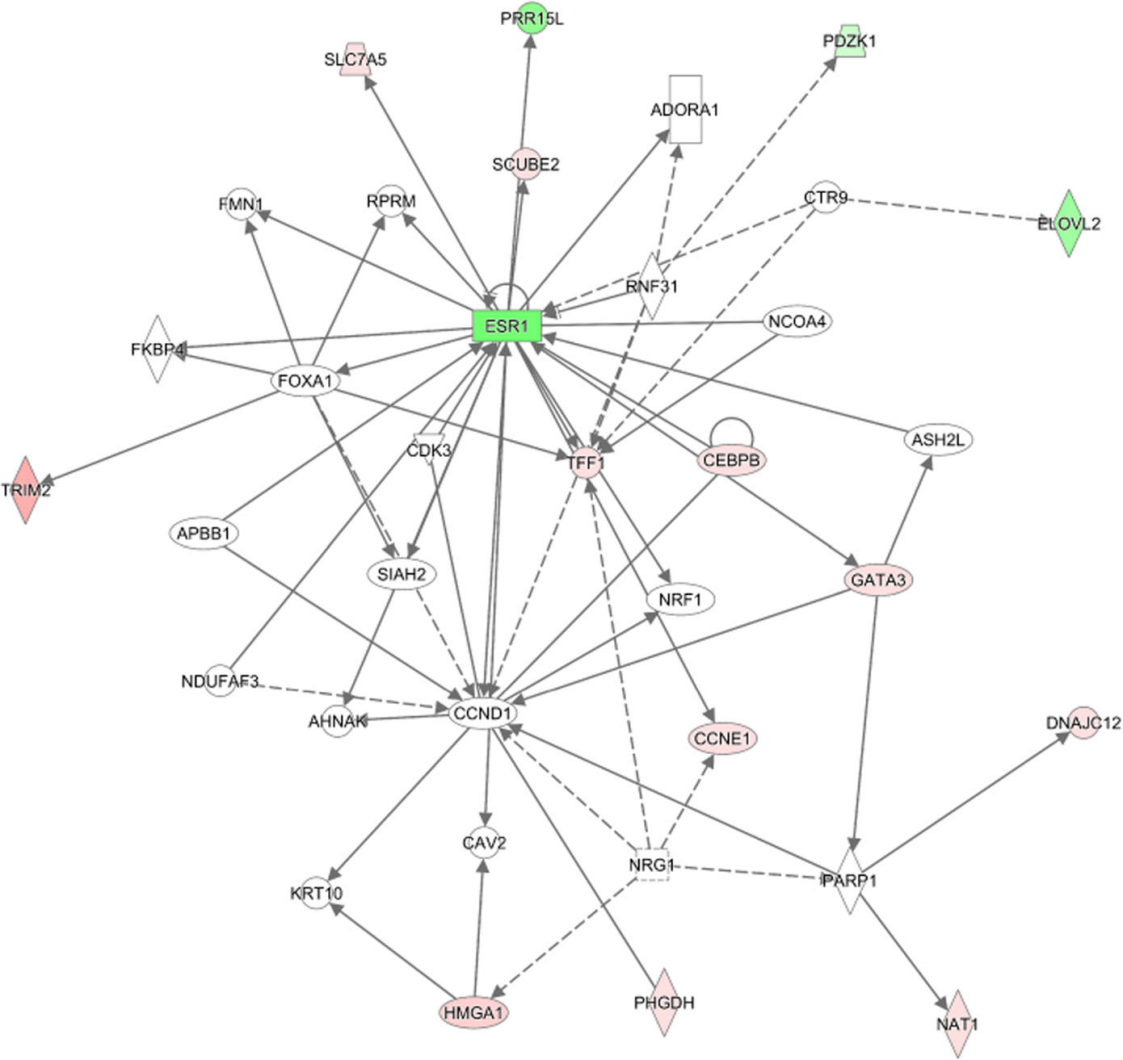
The largest IPA gene network corresponding to the largest gene cluster of MTM for the breast cancer dataset. Red/Green denote genes which have increased/decreased expression among relapsing patients compared with non-relapsing patients. Solid/Dashed lines denote direct/indirect interactions. Genes highlighted with magenta borders are identified with cellular development, cellular growth, and cell cycle functions. ESR1 and CCND1 seem to be interacting with each other as well as many other genes whose interactions are affected in breast cancer. The faded colors denote smaller fold changes. As many genes involved in breast cancer relapse are heterogeneous with a small sub-population heavily affected, fold changes might fail to grasp the extent of the gene effect

Considering a weighted graph where nodes are genes and edge weights are − logp-value of the gene pair, we observed many detected gene pairs construct highly connected clusters. To verify if the selected gene pairs are biologically relevant we (a) associated each gene with its smallest gene pair *p*-value, (b) selected the top 200 genes, (c) constructed their graph, (d) used community detection algorithm of [43] to identify network clusters, (e) selected the genes corresponding to the largest cluster, and (f) used Ingenuity Pathway Analysis^1^ (IPA) [44] to identify the networks associated with these genes only using experimentally observed results as well as the top canonical pathways. The top canonical pathways and the largest detected network are provided in Figs. 12 and 13, respectively. Note the top ranking IPA gene network is identified with cellular development, cellular growth, and cell cycle functions. The log fold changes, computed using method of [45], were used to identify over/under-expressed genes in the network; however, as data is highly heterogeneous, these effects might not be highly pronounced. Many of the selected genes are connected directly or indirectly with only one gene in between. Literature review suggests many of the top IPA pathways are also affected in breast cancer.

**Fig. 14:**
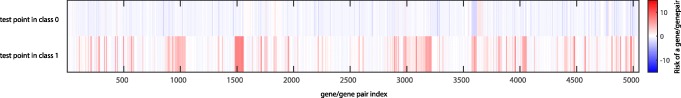
The associated un-normalized risk scores, i.e., S(X_f_) and $S(X_{f_{i},f_{j}})$, of two test points. Genes and gene pairs which are not used in the decision rule automatically get a risk of zero. Note most genes/gene pairs assign a larger risk to the point in class 1 (high risk patients) to the point in class 0 (low risk patient)

We now randomly leave out one point in each class, train LAS-HDMR, and look at the genes and gene pair that yield the highest scores in absolute values for these two test points. The minimum value for HDMR terms, either S(X_f_) or $S(X_{f_{i},f_{j}})$, was −*3*.*62* and −*0*.*59* for the points in classes 0 and 1, corresponding to gene pairs GREB1 and NAT1, and ZNF673 and ZNF391, respectively. The largest values were 3.64 and 14.32 respectively, corresponding to the ERBB2 gene, and gene pair ZNF395 and PTOV1. Figure 14 plots how the different genes and gene pairs are combined to arrive at the final log-likelihood ratio estimate.

### Lung cancer

**Fig. 15:**
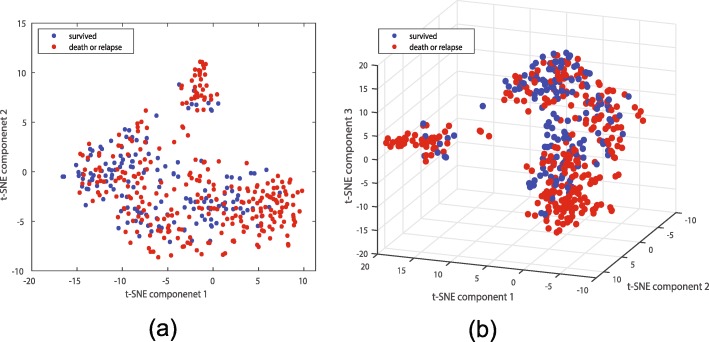
t-SNE plots of the lung cancer dataset separating the relapsing or dead (high risk) and surviving (low risk) patients: (**a**) 2D and (**b**) 3D plots. In both figures blue and red denote the surviving and relapsing/dead patients, respectively. Both t-SNE plots suggest there are at least two high risk subpopulations

Data obtained in [46] is deposited on GEO with accession number GSE68465, containing expressions of 443 lung cancer patients. 279 patients whose cancer relapsed or died within the follow up time comprise class 1, and the remaining 164 patients comprise class 0. This dataset is based on the GPL96 platform. We again only use probes mapping to genes, perform a log-normalization step, randomly select 250 points in class 0 and 140 points in class 1 for training, use the remaining points for testing, use the top 100 LRT genes for classifier design which we evaluate on test data, and iterate 100 times. Before we train the classifiers we provide 2D and 3D t-SNE [39] plots using the cityblock distance in Fig. 15, suggesting the two classes do separate; however, many patients who relapsed or died within six year (high risk) resemble those who survived (low risk). Both t-SNE plots suggest there are at least two high risk subpopulations.

Table 1 lists the AUCs (over test data), and Fig. 9b provides the ROC curves. Again we observe that none of the classifiers enjoy a very high AUC, and both LAS-HDMR and LABS-HDMR enjoy competitive performance compared with other classifiers. This may again suggest that a quadratic model might not be enough to capture the complicate structure of data. In this dataset, the variants of LAS-HDMR and LABS-HDMR achieving the highest AUCs are RLDA with *λ*=*0*.*1* and RLDA with *λ*=*0*.*2*, respectively. Figure 10b provides the Kaplan-Meier survivorship plots based on the assigned labels to the test data, averaging over 100 iterations for LAS-HDMR and LABS-HDMR, providing extra assurance that indeed the proposed algorithms separate the two classes. Here, the large ratio of high risk patients who resemble low risk ones in the t-SNE plots of Fig. 15 suggest that a reasonable decision rule would mislabel many high risk patients as low risk, explaining the low survivorship of the estimated low risk patients in Fig. 10b.

Table 5 lists the top 10 genes of LAS-HDMR and their associated risks. We again observe that many of the top genes, such as bromodomain PHD finger transcription factor (BPTF) [47] and LUC7 like 3 pre-MRNA splicing factor (LUC7L3) [48], are shown or suggested to be affected in lung cancer. Table 6 lists the top 10 gene pairs and their associated risks. A deeper analysis, including the results of LABS-HDMR, is provided in the Supplementary.

**Table 5:** Top lung cancer genes used for classification by LAS-HDMR

Rank	Gene	Risk	Rank	Gene	Risk
1	BPTF	2.5955	6	KIAA1033	1.5576
2	SEC63	2.4024	7	LUC7L3	1.398
3	UBXN4	1.7646	8	SON	1.3294
4	SRSF2IP	1.6672	9	PPIG	1.277
5	ATRX	1.5954	10	SF3B1	1.2601

**Table 6:** Top LAS-HDMR gene pairs for the lung cancer dataset

Rank	Gene 1	Gene 2	Risk
1	ATRX	DDX17	0.4683
2	ZEB1	BCLAF1	0.4663
3	IQGAP1	BPTF	0.4583
4	SON	UBXN4	0.4486
5	PRMT2	RUFY3	0.4433
6	ATP6V1G2, BAT1	SEC63	0.4402
7	LUC7L3	SMC5	0.4275
8	RBL2	MLL	0.3951
9	SRSF2IP	ENC1	0.3643
10	SPIN1	UBE2W	0.356

**Fig. 16:**
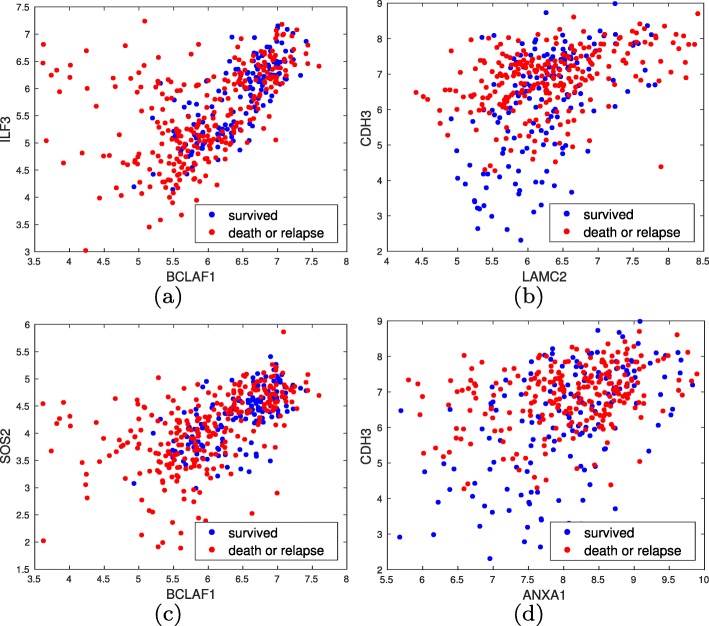
Scatter plots of gene pairs with significant interactions for the lung cancer dataset. Blue denotes patients who survived for at least sic years after diagnosis (low risk) and red denotes patients who died or relapsed within six years (high risk). It seems: (**a**) for a subpopulation of high risk patients BCLAF1 has decreased expression and ILF3 is over-expressed, (**b**) decreased expression of LAMC2 and CDH3 is an indicator of low risk, decreased expression of LAMC2 and over-expression of CDH3 is an indicator of “medium” risk, and over-expression of both is an indicator of high risk, (**c**) decreased expression of BCLAF1 or SOS2 compared with each other is an indicator of high risk, and (**d**) decreased expression of CDH3 is an indicator of low risk, over-expression of CDH3 and low expression of ANXA1 is an indicator of high risk, and over-expression of both results in medium risk

**Fig. 17:**
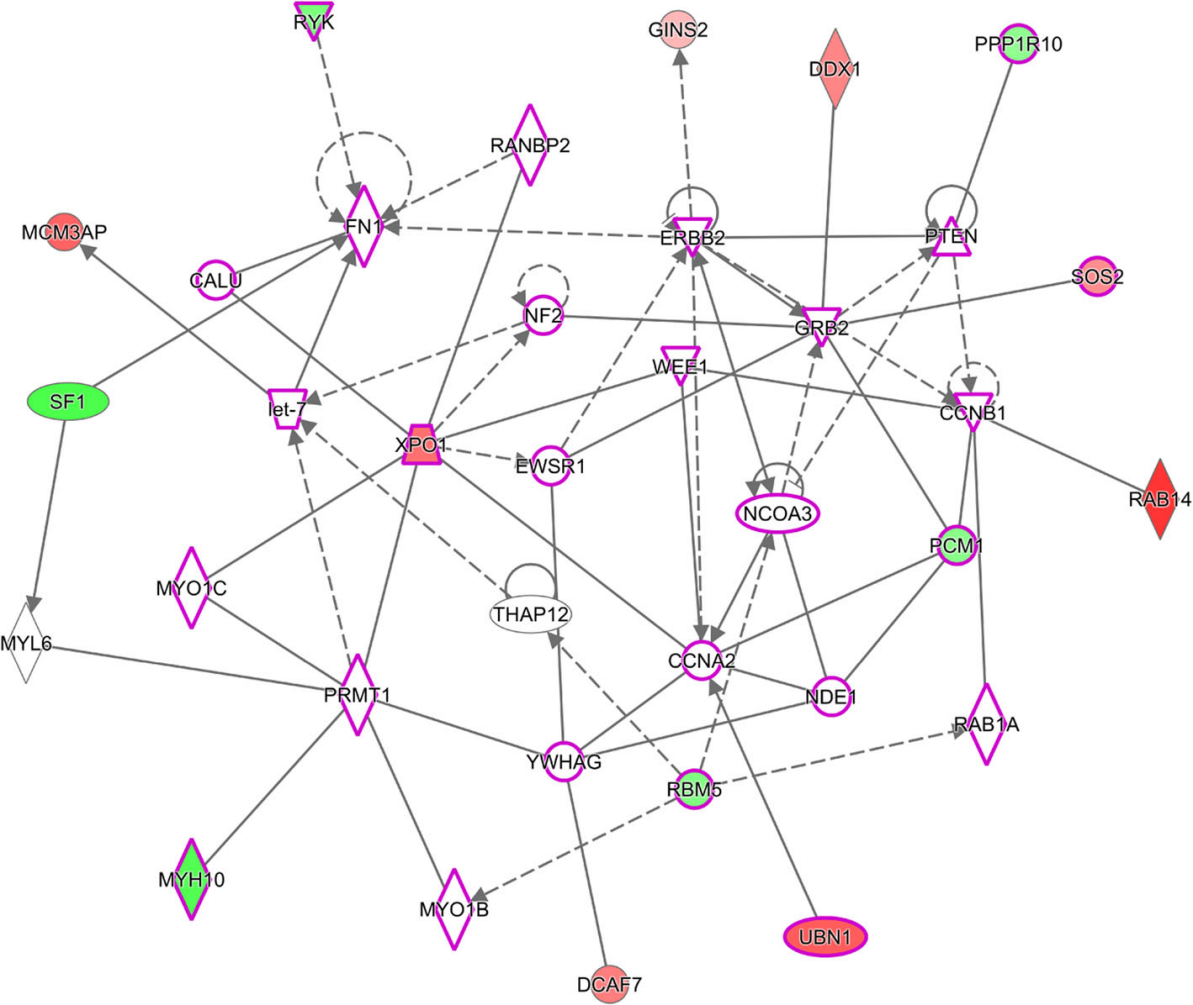
The largest IPA gene network corresponding to the largest gene cluster of MTM for the lung cancer dataset. Green/Red denote decreased/increased expression among high risk patients compared with low risk patients. Solid/Dashed lines denote direct/indirect interactions. Genes highlighted with magenta borders are associated with cell cycle, cellular assembly and organization, and cellular function and maintenance. Many dysregulated gene pairs seem to be affected via a mediating gene. Note the effect of each individual gene is more pronounced than the breast cancer dataset

For each gene we only use the probe ranking highest by LRT giving us 13,211 different genes and 8,758,655 pairwise dependence tests. We also perform outlier detection to further improve identifying general gene interaction patterns. Bounding FDR by *5%* using BH 701410 gene pairs are significant, about *0*.*8%* of all tests. Table 7 lists several of the top gene pairs and their adjusted *p*-values. Figure 16 provides scatter plots. We again observe MTM detects interesting pairwise interactions: (a) It seems there is a subpopulation of high risk lung cancer patients with poor survival for whom BCL2 associated transcription factor 1 (BCLAF1) is under-expressed and interleukin enhancer binding factor 3 (ILF3) is over-expressed. For other patients, irrespective of their label, these two genes are positively correlated. (b) Patients for whom both laminin subunit gamma 2 (LAMC2) and cadherin 3 (CDH3) are over-expressed have a high chance of relapse/death, those for whom CDH3 is over expressed and LAMC2 is under-expressed have a “medium” chance of relapse/death, and those for whom both CDH3 and LAMC2 are under-expressed have a low chance of relapse/death. (c) Under expression of either BCLAF or SOS Ras/Rho guanine nucleotide exchange factor 2 (SOS2) can be used as an indicator of poor survival. (d) Patients for whom both Annexin A1 (ANXA1) and CDH3 are over-expressed have a medium chance of relapse/death, those for whom CDH3 is over expressed and ANXA1 is under-expressed have a high chance of relapse/death, and those for whom both CDH3 and ANXA1 are under-expressed have a low chance of relapse/death. Finally, we again perform the IPA analysis similar to the breast cancer dataset, expect we include highly probable interactions in the analysis as well as experimentally observed ones. Figure 17 plots the detected network, which is associated with cell cycle, cellular assembly and organization, and cellular function and maintenance. Again observe many of the selected genes are connected with at most two genes in between. A deeper analysis is provided in the Supplementary.

**Table 7:** Top lung cancer gene pairs and adjusted *p*-values (×*10*^−*8*^)

Rank	Gene 1	Gene 2	adj *p*-value
1	BCLAF1	ILF3	<*10*^−*4*^
2	CDH3	CST6	0.0001
3	LAMC2	CDH3	0.0001
4	CDH3	PLAU	0.0006
5	S100A10	CDH3	0.0014
6	SMARCC1	BCLAF1	0.0015
7	BCLAF1	PCM1	0.004
8	ITGA3	CDH3	0.004
9	KRT19	CDH3	0.0064
10	BCLAF1	UBN1	0.0088

## Discussion

In the simulations and real data analyses we observed that both LAS-HDMR and LABS-HDMR enjoy competitive prediction accuracies compared with several popular classification rules. Additionally, they explicitly reveal how individual features or pairwise feature interactions motivate certain decisions, and how unique patterns of a new observation motivate its predicted label. Scatter plots of breast and lung cancer gene pairs in Figs. 11 and 16, respectively, suggest a quadratic classifier is adequate for predicting class labels from each gene pair; however, AUCs are not very high. This suggests higher order expansions, i.e., the joint interaction of three genes or more, are necessary to increase prediction accuracy. In these datasets we observe LABS-HDMR assigns larger risks to the top gene pairs compared with individual genes (see Tables 2 and 3 for breast cancer and Tables 5 and 6 for lung cancer), suggesting gene interactions are crucial to reliable prediction; not that linear classifiers miss important information in the data, but that pairwise interactions seem to carry more information about class labels than individual genes. In the leukemia dataset (studied in the Supplementary) we observed both LAS-HDMR and LAS-HDMR perform competitively, but all methods seem to enjoy high AUCs (AUC was larger than *94%* for all classifiers). In particular, we observed highest AUCs for linear classifiers, suggesting there is no need to use more complex rules. In particular, quadratic rules such as RQDA perform inferior to LAS-HDMR and LABS-HDMR. Finally, in the leukemia dataset we observe gene pairs have much smaller risks compared with individual genes.

MTM is an interesting test, allowing to extract co-expression patterns that differ between the two classes, i.e., identify pairwise interactions that differ between the two classes. MTM efficiently takes advantage of information in the data and is computationally fast. This is in contrast to several recent pipelines proposed for analyzing gene-pairs that are rather computationally intensive or may rely on large datasets (see [4, 5, 7–10] for examples).

## Conclusion and future work

Glass box models for binary classification open many avenues of research for analyzing genomic data as they enable us to make meaningful hypotheses about the underlying biological dysfunctions that are involved in a disease. To that end, HDMR seems to be an interesting theory for studying low dimensional glass-box models. The limitation of this approach in its current form is the assumption of known mechanisms that output the log-likelihood ratios given any partial observation. On the other hand, different feature sets can use different classification rules tailored to their joint distribution. This provides HDMR with tremendous flexibility, for instance, we may use QDA for some feature while a GLM is used for another feature pair. While this is a very exciting potential benefit of HDMR, we did not really exploit it in our current analysis and leave its careful consideration for possible future work. Another future direction for improving LAS-HDMR and LABS-HDMR is the development of more efficient methods of parameter selection to reduce computation cost of the currently implemented grid search approach.

The ability of MTM to identify pairwise interactions containing information not encoded in the likelihood function of each feature is a very practical contribution of our work. Here, instead of determining if two features are dependent, the goal is to verify if the pairwise interactions contain additional information about the classes which no model can extract by observing features individually. To do so, we developed a test where the null assumes the first order HDMR expansion is sufficient to explain the class differences. In the case of Gaussian features this translates into testing specific structures on the covariance matrices. Synthetic simulations and experimental data analyses suggest that MTM is indeed a powerful tool to extract dysregulated pairwise gene co-expression patterns that motivate new hypotheses about cancer biology. In the real data analyses we observed many pairs might have a gene in common, for instance, SPDEF in the breast cancer dataset and BCLAF1 and CDH3 in the lung cancer dataset. These patterns motivate hypotheses not only about gene pairs, but more generally about heavily correlated marker families. Graph representations are interesting tools for analyzing families of pairwise interactions, and graph community detection algorithms can infer marker interaction blocks given pairwise interaction graphs. However, future work should investigate the use of HDMR for directly inferring such structures. To that end, LABS-HDMR seems to be an ideal first step, where its constructed blocks can be potential first approximations to marker families of interest.

## Supplementary information

**Additional file 1** Supplementary. The supplementary contains extra information regarding the synthetic simulations and real data analysis. It also studies a Leukemia dataset not discussed in the main manuscript.

## Data Availability

All cancer data used in this work is publicly available via GEO database accession numbers GSE25066 and GSE68465. The synthetic datasets are available upon request.

## Abbreviations

AGR2: Anterior gradient protein 2 homolog
ANXA1: Annexin A1
AUC: Area under the ROC curve
BCLAF1: BCL2 associated transcription factor 1
BH: Benjamini-Hochberg step-up procedure
BPTF: Bromodomain PHD finger transcription factor
CDH3: Cadherin 3
CPB1: Carboxypeptidase B1
CXCL8: C-X-C motif chemokine ligand 8
FDR: False discovery rate
GATA3: GATA binding protein 3
GEO: Gene expression omnibus
GLM: Generalized linear model
GREB1: Growth regulating estrogen receptor binding 1
HDMR: High dimensional model representation
ILF3: Interleukin enhancer binding factor 3
IPA: Ingenuity pathway analysis
kNN: k nearest neighbors
LABS-HDMR: Linear approximation of block second order HDMR expansion
LAMC2: Laminin subunit gamma 2
LAS-HDMR: Linear approximation second order HDMR expansion
LASSO: Least absolute shrinkage and selection operator
LDA: Linear discriminant analysis
LRT: Likelihood ratio test
LUC7L3: LUC7 like 3 pre-MRNA splicing factor
MSE: Mean squared error
MTM: Multiple test mixing for pairwise interactions
NAT1: N-Acetyltransferase 1
QDA: Quadratic discriminant analysis
RF: Random forest
ROC: Receiver operator characteristic
RLDA: Regularized linear discriminant analysis
RQDA: Regularized quadratic discriminant analysis
SLC2A10: Solute carrier family 2 member 10
SOS2: Ras/Rho guanine nucleotide exchange factor 2
SPDEF: SAM pointed domain containing ETS transcription factor
SCUBE2: Signal peptide CUB domain EGF-like 2
SLAC2A: Synaptotagmin-like protein 2A
SVM: Support vector machine
TCGA: The cancer genome atlas

## Footnotes

1 QIAGEN Inc., https://www.qiagenbioinformatics.com/products/ingenuity-pathway-analysis
