# Supplementary material for: High dimensional model representation of log-likelihood ratio: binary classification with expression data
Source: BMC Bioinformatics. 2020 Apr 25;21:156. doi: 10.1186/s12859-020-3486-x (PMC7183128; doi:10.1186/s12859-020-3486-x)
Supplement: Supplementary file 1 — Additional file 1 Supplementary. The supplementary contains extra information regarding the synthetic simulations and real data analysis. It also studies a Leukemia dataset not discussed in the main manuscript. [file 12859_2020_3486_MOESM1_ESM.pdf]

RESEARCH

High dimensional model representation of log likelihood ratio: Binary classification with expression data (Supplementary)

Ali Foroughi pour[1,2], Maciej Pietrzak[3], Lori A Dalton[1] and Grzegorz A. Rempała[2,4*]

*Correspondence:
rempala.3@osu.edu
[4]College of Public Health, 250 Cunz Hall, 1841 Neil Ave., 43210 Columbus, USA
Full list of author information is available at the end of the article
†Equal contributor

1 Synthetic Simulations

Here we provide the AUCs of synergetic and marginal markers with equal and unequal correlations, as well as their ROC plots for $n = 60$. Figures 1 and 2 plot the AUC and ROC curves of all the 4 tested marker distributions. Note the results for synergetic markers is similar to marginal markers. Additionally, for $n = 60$ LAS-HDMR and LABS-HDMR have competitive AUC compared with other tested methods, and perform superior to all other tested classification algorithms when data has unequal correlations between the two classes. In settings with equal correlations RLDA and linear probit, which are both linear models, perform superior to all other methods, and are followed by LAS-HDMR and LABS-HDMR.

1.1 The Problem of Sample Size

Many clinical trials aim to predetermine a minimum sample size necessary to arrive within a certain distance of the true AUC with a high probability. Various methods with different modeling assumptions are proposed for this task [1, 2, 3]; however, most of these standard methods, that also enjoy closed form solutions, are typically suitable for univariate problems. Given the distribution parameters these methods usually compute the AUC and its variance, and use an asymptotic normal approximation to compute the minimum necessary sample size. Refer to [1] for additional examples.

Several extensions for multivariate problems have been proposed [4, 5], which are typically not in closed form, rely on Monte Carlo methods, and may be computationally intensive. Here we only compute the AUC's for LDA, QDA, and an "informed" classifier, and compare these (asymptotic) results with the AUC's we got from the simulation. It is straightforward to show that the covariance matrix of a marker block in class 1 is

$$\Sigma^H = 0.5(\Sigma_0 + \Sigma_1) + 0.25\mu_1 \times \mu_1^T. \quad (1)$$

Additionally, the mean vector of a block of heterogeneous markers in class 1 is $0.5\mu_1$. Recall that we already know the distribution parameters of markers in class 0. We use the true mean and covariances of each block to construct the true mean vector and covariance matrix of all markers, which we use to train the classifiers. We then generate a random training sample with 1000 points in each class to compute the AUC's, averaging over 500 iterations. Additionally, it is straightforward

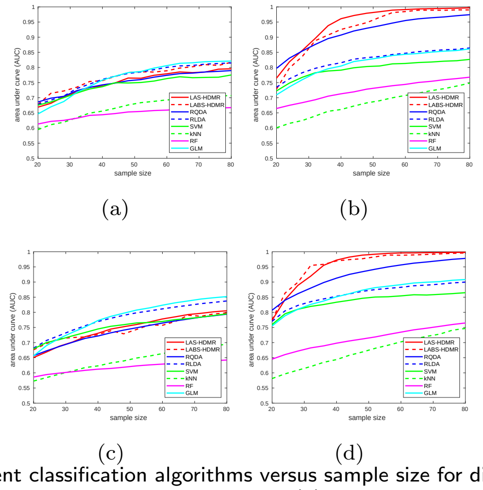

Figure 1 AUC of different classification algorithms versus sample size for different data generation models: (a) synergetic features and equal correlations, (b) synergetic features and unequal correlations, (c) marginal features and equal correlations, and (d) marginal features and unequal correlations

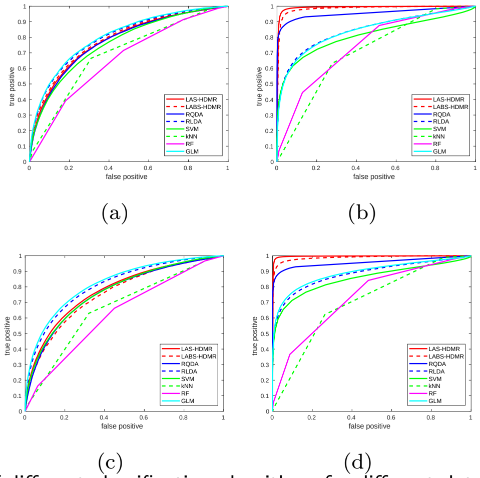

Figure 2 ROC curve of different classification algorithms for different data generation models when $n = 60$: (a) synergetic features and equal correlations, (b) synergetic features and unequal correlations, (c) marginal features and equal correlations, and (d) marginal features and unequal correlations

Table 1 Asymptotic AUC of several classification rules

classification rule	QDA	LDA	Informed
synergetic, equal correlations	90.05%	90.41%	>99%
synergetic, unequal correlations	99.93%	93.63%	>99.99%
marginal, equal correlations	90.59%	89.35%	>99%
marginal, unequal correlations	99.93%	92.65%	> 99.99%

to compute the AUC of a an "informed" classifier that correctly assumes there are two subpopulations, which heterogeneous markers have distributional differences in each subpopulation, what the exact distribution parameters are, and solves the three class problem: class 0, class 1 subpopulation 1, and class 1 subpopulation 2. Table 1 lists the computed asymptotic AUC of each classifier. These results suggest the classes are well separated and it is possible to obtain large AUC's given a large sample size. On the other hand, for this setting, we obtain much smaller AUC's in the simulations due to the small training sample sizes.

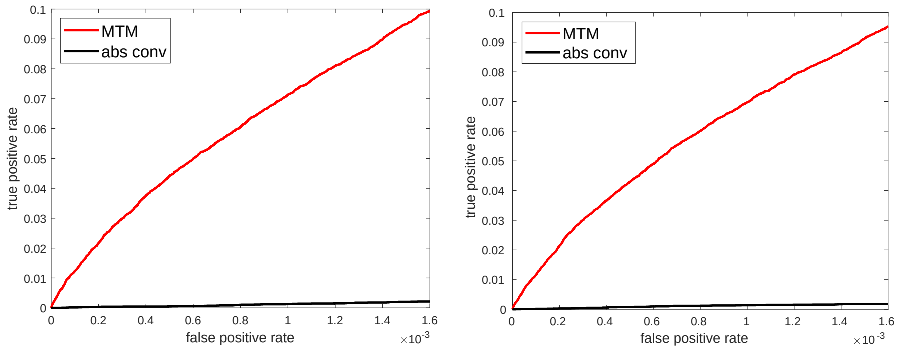

(a) (b)

Figure 3 ROC curves of MTM and abs conv for detecting pairwise feature interactions across different marker types with equal correlation coefficients when $n = 40$: (a) synergetic markers and (b) marginal markers.

1.2 Evaluation of MTM over synthetic data

Here we compare MTM with the algorithm proposed in [6] for detecting pairwise gene synergies, here after called absolute conversion (abs conv). Given the literature review and the results provided in [6] we believe abs conv would enjoy superior performance compared with other algorithms proposed for detecting pairwise interactions in the literature, while balancing computation cost. Figure 3 provides a comparison of MTM and abs conv averaging over 50 iterations for marginal and synergetic markers with equal correlations ($n = 40$). In both settings MTM outperforms abs conv. We also observed that in these simulations MTM is about two orders of magnitude faster than abs conv; however, it may be due to a efficient implementation of internal components of MTM as built-in MATLAB functions.

2 Real Data

Here we study the breast and lung cancer datasets in more detail, and study a leukemia dataset as well.

2.1 Breast Cancer

Data obtained in [7, 8] is deposited on gene expression omnibus (GEO) [9] with accession number GSE25066, containing expression levels of 397 relapse free and 111 relapsing breast cancer patients. Data is based on the GPL96 platform, and is already pre-processed and normalized. The dataset contains 22,283 probes, of which 20,967 map to genes. We only use probes that map to genes in our analysis.

We first randomly select 100 relapsing patients and 360 non-relapsing patients as training, and the remaining points are used for testing. We then use the likelihood ratio test (LRT) of [10] to pick the top 100 genes. The training data of these genes is used to train the classifiers of the main paper, and their performance is evaluated on the test data. We iterate 100 times. Our choice to use LRT to select top genes instead of the intrinsic ability of HDMR expansion is two fold: (1) LRT is computationally much less intensive and helps with reducing the computational burden, (2) a priori we do not know if marker genes have equal or unequal variances in both classes. Therefore, we use the selection algorithm that can capture differences in variances

Table 2 AUC and portion of variance explained of the classification algorithms for the breast cancer dataset

method	LAS-HDMR	LABS-HDMR	RQDA	RLDA	SVM	RF	kNN	GLM
AUC	64.21%	69.95%	62.70%	66.04%	55.34%	60.87%	58.18%	67.55%
por. var. expl.	78.81%	80.46%	77.49%	78.58%	78.30%	78.27%	78.26%	78.91%
por. bal. var. expl.	63.61%	68.28%	62.66%	65.48%	54.54%	60.18%	56.29%	64.98

Table 3 Top breast cancer genes used for classification by LAS-HDMR

Rank	Gene	Risk Amount	Rank	Gene	Risk Amount
1	ORM1, ORM2	0.92	6	ACADSB	0.75
2	IL8	0.87	7	PTOV1	0.75
3	ZNF395	0.85	8	ZNF673	0.75
4	GREB1	0.8	9	AR	0.74
5	TBC1D9	0.78	10	LGALS8	0.71

as well. Note that if no differences in variances exist then we only look at differences in the means by looking at LRT, and we would expect it to perform similar to t-test. A more detail discussion is provided in [11]. Finally, we have included RLDA as a potential classifier as well, in case LRT select genes based on differences in the means not variances.

Table 2 lists the AUC of different classifiers on this dataset, as well as the portion of variance they explain. Note for binary class labels, of the portion of variance explained reduces to $1 - p_e$, where p_e is probability of error, either miss or false alarm. Note that as sample is imbalances in class zero, false alarm dominates the probability of error. Hence we also report proportion of balanced variance explained, being the average of probability of miss and false alarm, i.e., pretending as if the sample is balanced.

As Tab. 2 suggests, all methods do not enjoy a high AUC, suggesting 100 features might not be sufficient to separate the two classes. We observed that the variant of LAS-HDMR using RQDA with $\lambda = 0.8$ achieved the highest AUC. The largest AUC for the variant of LAS-HDMR using RLDA was 63.12% obtained for $\lambda = 0.9$. In contrast, LABS-HDMR seems to enjoy the highest AUC, obtained using RQDA with $\lambda = 0.1$, which is the closest tested variant to conventional QDA. However, even this AUC is not very high. This may suggest that a second order expansion is not satisfactory enough for this data, and we need to look at higher order expansions and more complex models for this dataset.

Although LAS-HDMR and LABS-HDMR may not yield high classification accuracy, similar to other classification algorithms, their glass box nature simplifies the process of identifying genes and gene pairs that contribute the most to the classifier's prediction. We use all of the data for training, and use RQDA with $\lambda = 0.8$ to obtain the log likelihood ratios. Table 3 lists the top 10 genes of LAS-HDMR. Note many of the top genes, such as IL8 [12, 13, 14] and GREB1 [15, 16, 17] are shown to be affected in breast cancer. We plot the histogram of top four LAS-HDMR genes in Fig. 4. As the figure suggests, these genes do seem to have distributional differences across the two classes, and LAS-HDMR correctly assigns them a non-zero weight. As we have already performed a first phase filtration reporting the top 100 features, the fact that top LAS-HDMR genes are high-profile markers is not unexpected, but we now also can estimate how much each feature affect the risk of relapse on average. Note LAS-HDMR shines as it can similarly depict which gene interactions are important the most.

Table 4 lists the top 20 gene pairs selected by LAS-HDMR, and Fig. 5 provides the scatter plots for several gene pairs. Comparing Tabs. 3 and 4 we observe that

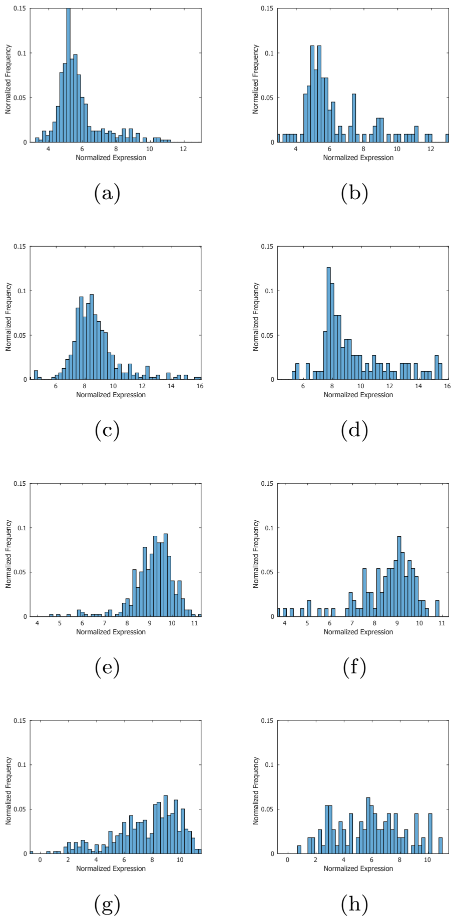

Figure 4 Histogram of breast cancer genes ranking high by LABS-HDMR: (a) non-relapsing ORM1, ORM2 and (b) relapsing ORM1, ORM2, suggesting ORM1 and ORM2 are over-expressed in a subpopulation of relapsing patients, (c) non-relapsing IL8 and (d) relapsing IL8, suggesting IL8 is over-expressed in a subpopulation of relapsing patients, (e) non-relapsing ZNF395 and (f) relapsing ZNF395, suggesting ZNF395 has lower expression in a subpopulation of relapsing patients, and (g) non-relapsing GREB1 and (h) relapsing GREB1, suggesting GREB1 has lower expression in a subpopulation of relapsing patients.

gene interactions tend to have a larger risk than individual features. As Fig. 5 suggests, these gene pairs seem to be affected differently between the two classes, and their interactions seem to be different comparing relapsing versus non-relapsing breast cancer patients. Being able to explicitly observe the gene-gene interactions that seems to be dis-regulated the most between the two classes may be the most interesting property of LAS-HDMR, as it extremely helps to gain insight on the underlying biological mechanisms. For example, in Fig. 5(b) we observe that in non-relapsing patients either GREB1 or CPB1 are over-expressed, and we need under-expression of both GREB1 and CPB1 to have a high risk of relapse. Finally, note many of the top gene-gene interaction pairs contain GREB1, SCUBE2, GATA3, and IL8, suggesting their interaction might be key to studying breast cancer.

LAS-HDMR also assigns very little risk to many gene pairs, suggesting they have little interactions, and that a first order expansion is sufficient to grasp their effect. Figure 6 provides the scatter plot of several gene pairs that have small risks, which

Table 4 Top gene pairs with largest risks of the breast cancer dataset

Rank	Gene 1	Gene 2	Risk Type	Risk Amount
1	GREB1	SCUBE2	increasing	2.24
2	GREB1	CPB1	increasing	2.23
3	ORM1, ORM2	GREB1	increasing	2.22
4	GREB1	IL8	increasing	2.22
5	ZNF395	GREB1	increasing	2.1
6	GREB1	GATA3	increasing	2.07
7	GREB1	NAT1	increasing	2.06
8	GREB1	TBC1D9	increasing	2.05
9	GREB1	ACADSB	increasing	2.03
10	ORM1, ORM2	SCUBE2	increasing	2.02
11	GREB1	AR	increasing	2.00
12	GATA3	SCUBE2	increasing	1.99
13	IL8	SCUBE2	increasing	1.99
14	LGALS8	GREB1	increasing	1.97
15	CPB1	SCUBE2	increasing	1.96
16	ZNF673	GREB1	increasing	1.96
17	PTOV1	GREB1	increasing	1.96
18	ACADSB	SCUBE2	increasing	1.92
19	FAM174B	GREB1	increasing	1.91
20	CCPG1	GREB1	increasing	1.89

are also removed by T_2 in parameter tuning. As the figure suggests, we do not observe an interesting interaction for these gene pairs.

We now study LABS-HDMR in more detail. The variant of LABS-HDMR that achieved the highest AUC used RQDA with $\lambda = 0.1$. Hence, we use all of training data with this variant to measure the risks of genes and gene-gene interactions. Table 5 lists the risk associated to the top 10 genes of LABS-HDMR, and Fig. 7 provides the histogram of the expressions of several top genes across the two classes. Again, we observe that these genes have distributional differences across classes, the risk associated to each gene by LABS-HDMR is provided in Tab. 5, and many of these top genes, such as GMPR2 [18, 19] and APC [20, 21, 22], are already shown to be affected in breast cancer. Note by using a smaller value for regularization of RQDA (1) the top 10 genes are affected and (2) we tend to assign larger risks to individual genes.

We now study the top gene pairs. The top 20 gene pairs with largest risks are provided in Tab. 6, and the scatter plot of several gene pairs are provided in Fig. 8. We again observe that gene pairs with highest risks may have interesting interaction patterns that motivate further biological verification; however, as RQDA is using less regularization, it is more affected by points that are far from the majority of points in their class. Hence, larger regularization might be more suitable when studying gene pair interactions, although it may not yield the best classification results. For instance, in Fig. 8(f) we observe that (1) low expression of APC and high expression of MORC3 is an indicator of high chance or relapse, (2) low expression of MORC3 and high expression of APC is an indicator of low chance of relapse, and (3) high expression of both genes is not very informative. However, the observed patterns are not as dominant as for the LAS-HDMR case which used larger regularization. Furthermore, in contrast to LAS-HDMR, LABS-HDMR assigns large risks to gene pairs that are heavily affected for small subpopulations, due to the built in averaging property, merging the less prevalent patterns to arrive at a normalized risk for each block.

Now we look for significant pairwise gene interactions. First, for each gene, we only consider the probe ranking highest by LRT for that gene, so that probes mapping to the same genes do not disrupt the analysis. This way we get 13211

Table 5 Top breast cancer genes used for classification by LABS-HDMR

Rank	Gene	Risk Amount	Rank	Gene	Risk Amount
1	GMPR2	3.1302	6	TOX4	2.2306
2	IGBP1	2.9628	7	ACTR1A	2.2104
3	APC	2.7936	8	CAB39L	2.1352
4	KIAA0319L	2.6882	9	TFAM	2.0782
5	MAN1A2	2.3862	10	SNX5	2.0652

Table 6 Top gene pairs used for classification by LABS-HDMR on the breast cancer dataset

Rank	Gene 1	Gene 2	Risk Type	Risk Amount
1	TFAM	APC	increasing	1.9419
2	VPS13A	APC	increasing	1.9069
3	TFAM	TOX4	increasing	1.8517
4	TOX4	APC	increasing	1.8432
5	YIPF5	APC	increasing	1.825
6	GMPR2	APC	increasing	1.813
7	ACTR1A	APC	increasing	1.7973
8	TFAM	YIPF5	increasing	1.7855
9	TFAM	GMPR2	increasing	1.7546
10	YIPF5	TOX4	increasing	1.7544
11	TRIM3	APC	increasing	1.7428
12	MORC3	APC	increasing	1.7325
13	APH1B	APC	increasing	1.7246
14	NAPA	APC	increasing	1.7223
15	THUMPD1	APC	increasing	1.713
16	ZNF395	APC	increasing	1.7045
17	NPRL3	TOX4	increasing	1.6952
18	SNX5	APC	increasing	1.6658
19	CPOX	APC	increasing	1.6631
20	THUMPD1	TOX4	increasing	1.6594

different genes, which give us 87258655 different pairwise dependence tests. We also observed that MTM can be heavily affected by small subpopulations, heavy tails, and outliers. Therefore, we use MATLAB's built in `isoutlier` function with its default values to remove outliers before further analysis. Bounding FDR by 5% we observed that 1275351 pairwise interactions are significant, being about 1.46% of tested hypotheses. Table 7 lists the top 25 gene pairs and their adjusted p-values. Figure 11 provides scatter plots of several gene pairs. We observe many interesting patterns that require further investigation. For instance, it seems (1) over-expression of both SCGB1D2 and SCGB2A2 reduces the chance of breast cancer relapse, (2) under-expression of TFF3 and SPDEF increases the chance of relapse, (3) over-expression of MSN and under-expression of SPDEF increases the chance of relapse.

2.1.1 Network Analysis Results

Here we provide further details regarding the IPA results. Recall we first associated each gene with the smallest p-value of MTM test for all of it possible gene pairs. In other words, for gene g we set

$$p(g) = \min_{g' \neq g} P_{MTM}(g, g'), \tag{2}$$

where $P_{MTM}(g, g')$ is the p-value of MTM for gene pair g, g'. We then rank genes based on $p(g)$'s, and pick the top 200 genes with smallest p-values. Afterwards, we consider a graph where the selected genes are the nodes, and significant pairwise interactions denote the existence of an edge between nodes. The weight of an edge connecting genes g and g' is set to $-\log(P_{MTM}(g, g'))$. We then use the graph community detection algorithm of [23] to cluster genes to communities, partitioning genes to 4 disjoint communities. Figure 12 plots the largest detected community. We

Table 7 Top 25 gene pairs of the breast cancer dataset

Rank	gene 1	gene 2	adj P-value (col $\times 10^{-25}$)
1	SPDEF	MLPH	$< 10^{-4}$
2	MSN	SPDEF	$< 10^{-4}$
3	SCGB2A2	SCGB1D2	$< 10^{-4}$
4	TFF3	SPDEF	$< 10^{-4}$
5	RHOB	SPDEF	$< 10^{-4}$
6	CA12	SPDEF	0.0001
7	FBP1	SPDEF	0.0003
8	KRT18	SPDEF	0.0003
9	XBP1	SPDEF	0.0011
10	AGR2	NAT1	0.0011
11	SLC44A4	SPDEF	0.0014
12	SPDEF	FAM174B	0.0064
13	FOXA1	NAT1	0.0128
14	GATA3	SPDEF	0.0477
15	TSPAN1	SPDEF	0.0477
16	GLS	SPDEF	0.0478
17	ESR1	SPDEF	0.052
18	LASS6	SPDEF	0.052
19	TMBIM6	SPDEF	0.0794
20	TBC1D9	SPDEF	0.0794
21	PRNP	SPDEF	0.094
22	SPDEF	SLC2A10	0.0997
23	SPDEF	MYO5C	0.1032
24	SPDEF	TSPAN13	0.1032
25	NCK1	SPDEF	0.1112

then use the genes corresponding to this community for Ingenuity Pathway Analysis [1] (IPA) [24] , only using experimentally validated and strong interactions. IPA detects 11 networks, where the top network is provided in the main paper. Finally, Fig. 13 provides the top IPA pathways corresponding to this gene cluster.

2.2 Lung Cancer

Data obtained in [25] is deposited on GEO with accession number GSE68465, containing expression levels of 443 lung cancer patients. The 279 patients whose cancer relapsed within the follow up time or died comprise class 1, and the remaining 164 patients comprise class 0. This dataset is again based on the GPL96 platform containing 22,283 probes, of which 20,967 map to genes. We only use probes that map to genes in our analysis, perform a log-normalization, and use the top 100 LRT genes for analysis.

We randomly select 250 points in class 0 and 140 points in class 1 for training, and the remaining points are used for testing. We again iterate 100 times. Table 8 lists the AUC of each of the classifiers. Again we observe that none of the classifiers enjoy a very high AUC, and both LAS-HDMR and LABS-HDMR enjoy competitive performance compared with other classifiers. This may again suggest that a quadratic model might not be enough to capture the complicate structure of data. In this dataset, the variants of LAS-HDMR and LABS-HDMR achieving the highest AUC's are RLDA with $\lambda = 0.1$ and RLDA with $\lambda = 0.2$, respectively.

Table 9 lists the top 10 genes of LAS-HDMR and their associated risks, and Fig. 14 provides the histogram of some of the top genes. We again observe that many of the top genes, such as BPTF [26, 27, 28] and LUC7L3 [29], are shown or suggested to be affected in lung cancer. Table 10 lists the top 20 gene-gene interactions and their associated risks, and scatter plots of several gene pairs with large risks are provided in Fig. 15.

[1]QIAGEN Inc., https://www.qiagenbioinformatics.com/products/ingenuity-pathway-analysis

Table 8 AUC of the classification algorithms for the lung cancer dataset

method	LAS-HDMR	LABS-HDMR	RQDA	RLDA	SVM	RF	kNN	GLM
AUC	66.56%	67.67%	65.47%	66.62%	63.03%	66.87%	61.60%	68.22%
por. var. expl.	69.98%	70.77%	67.61%	69.60%	66.77%	72.25%	68.63%	71.22%
por. bal. var. expl.	64.77%	63.75%	62.78%	63.94%	59.92%	64.97%	59.06%	62.58

Table 9 Top lung cancer genes used for classification by LAS-HDMR

Rank	Gene	Risk Amount	Rank	Gene	Risk Amount
1	BPTF	2.5955	6	KIAA1033	1.5576
2	SEC63	2.4024	7	LUC7L3	1.398
3	UBXN4	1.7646	8	SON	1.3294
4	SRSF2IP	1.6672	9	PPIG	1.277
5	ATRX	1.5954	10	SF3B1	1.2601

Table 10 Top lung cancer gene pairs used for classification by LAS-HDMR

Rank	Gene 1	Gene 2	Risk Type	Risk Amount
1	ATRX	DDX17	increasing	0.4683
2	ZEB1	BCLAF1	increasing	0.4663
3	IQGAP1	BPTF	increasing	0.4583
4	SON	UBXN4	increasing	0.4486
5	PRMT2	RUFY3	increasing	0.4433
6	ATP6V1G2 BAT1	SEC63	increasing	0.4402
7	LUC7L3	SMC5	increasing	0.4275
8	RBL2	MLL	increasing	0.3951
9	SRSF2IP	ENC1	increasing	0.3643
10	SPIN1	UBE2W	increasing	0.356
11	PHF3	KIAA1033	increasing	0.3462
12	SF3B1	NAA15	increasing	0.3434
13	YTHDC1	TTC3	increasing	0.3311
14	SCAMP1	TOP1	increasing	0.3277
15	PRDM2	EEA1	increasing	0.3129
16	CLCN3	CEP350	increasing	0.3119
17	EIF5B	KIAA0776	increasing	0.308
18	MYH10	RFX7	increasing	0.304
19	PPIG	SOS2	increasing	0.3019
20	USO1	SETD2	increasing	0.3019

Table 11 Top lung cancer genes used for classification by LABS-HDMR

Rank	Gene	Risk Amount	Rank	Gene	Risk Amount
1	BPTF	2.0293	6	SON	1.2708
2	SEC63	1.7036	7	KIAA1033	1.2363
3	UBXN4	1.5221	8	LUC7L3	1.2337
4	ATRX	1.4856	9	RUFY3	1.1208
5	SRSF2IP	1.2834	10	SMC5	1.1004

We now study LABS-HDMR. Table 11 lists the top 10 genes and their associated risks, and Fig. 16 provides the histogram of some of the top genes. We again observe that many of the top genes are shown or suggested to be affected in lung cancer. Table 12 lists the top 20 gene-gene interactions and their associated risks, and scatter plots of several gene pairs with large risks are provided in Fig. 17. Finally, Fig. 18 provides the ROC curve of the lung cancer dataset.

Again for each gene we only use the probe ranking highest by LRT so that probes mapping to the same genes do not disrupt the analysis, and perform outlier detection. This way we get 13211 different genes, which give us 8758655 different pairwise dependence tests. Bounding FDR by 5% we observe 701410 gene pair interactions are significant, about 0.8% of all tests. Table 13 lists the top 25 gene pairs selected by each algorithm and their adjusted p-values. Figure 19 provides scatter plots of several gene pairs.

2.2.1 Network Analysis Results

Here we provide further details regarding the IPA results. We again selected the top 200 genes based on their $p(g)$'s, and consider the graph of selected genes and significant pairwise interactions where edge eights are $-\log(P_{MTM}(g, g'))$. We then use the graph community detection algorithm of [23] to cluster genes to communities,

Table 12 Top lung cancer gene pairs used for classification by LABS-HDMR

Rank	Gene 1	Gene 2	Risk Type	Risk Amount
1	MMP14	THRA	increasing	0.646
2	TRADD	HSPA6	increasing	0.6264
3	TRADD	THRA	increasing	0.624
4	TARDBP	TRADD	increasing	0.624
5	TRADD	MMP14	increasing	0.6235
6	TARDBP	THRA	increasing	0.6221
7	RPL30	TRADD	increasing	0.6202
8	PMS2P11	TRADD	increasing	0.6152
9	TARDBP	PMS2P11	increasing	0.606
10	MMP14	PTPN21	increasing	0.6018
11	PMS2P11	MMP14	increasing	0.5995
12	TRADD	PTPN21	increasing	0.5989
13	CYP2A6	HSPA6	increasing	0.5929
14	ATP6V1G2 BAT1	TRADD	increasing	0.5906
15	C1D	TARDBP	increasing	0.5872
16	ZNF146	TRADD	increasing	0.5854
17	THRA	HSPA6	increasing	0.5848
18	RPL28	TRADD	increasing	0.5848
19	C1D	MMP14	increasing	0.5813
20	MYST2	HSPA6	increasing	0.5779

Table 13 Top 25 gene pairs of the lung cancer dataset

Rank	gene 1	gene 2	adj P-value (col $\times 10^{-8}$)
1	BCLAF1	ILF3	0
2	CDH3	CST6	0.0001
3	LAMC2	CDH3	0.0001
4	CDH3	PLAU	0.0006
5	S100A10	CDH3	0.0014
6	SMARCC1	BCLAF1	0.0015
7	BCLAF1	PCM1	0.004
8	ITGA3	CDH3	0.004
9	KRT19	CDH3	0.0064
10	BCLAF1	UBN1	0.0088
11	CDH3	MAP7D1	0.0316
12	CDH3	FXYD5	0.0327
13	CDH3	KCNN4	0.0327
14	BCLAF1	SOS2	0.0327
15	CDH3	GPR87	0.0377
16	ATRX	MAP9	0.0387
17	BCLAF1	DCAF7	0.0409
18	BCLAF1	UBXN4	0.0426
19	PCM1	SOS2	0.0611
20	ANXA1	CDH3	0.0625
21	CDH3	SERPINB1	0.0663
22	GSTT1	CYB5A	0.1081
23	BCLAF1	CHD4	0.1491
24	BCLAF1	SON	0.156
25	MX1	CDH3	0.1993

giving us 5 disjoint communities. Figure 20 plots the largest detected community, containing 50 genes. We then use these genes IPA, only using experimentally validated and strong interactions. The top network is provided in Fig. 21. Finally, Fig. 22 provides the top IPA pathways corresponding to this gene cluster.

2.3 Leukemia Dataset

Data obtained in [30] is deposited on GEO with accession number GSE13204 containing expression levels of 74 healthy, 76 chronic myeloid leukemia (CML) patients, and expression levels of patients with other leukemia subtypes. Data is based on the GPL570 platform and is already pre-processed; however, here we further perform a log-normalization so that data is more suitable for the RLDA and RQDA classifiers. This dataset contains 54,765 probes, of which 42,450 probes map to genes. Again we only use probes mapping to genes in our analysis.

Table 14 Top CML genes used for classification by LAS-HDMR

Rank	Gene	Risk Amount	Rank	Gene	Risk Amount
1	IGLV2-23	662	6	IGJ	588
2	DUXAP10	642	7	IGH(A1,2-G1,3-V3,4)	582
3	IGHM	639	8	KIF21A	576
4	BLNK	625	9	EPB41L3	574
5	ITPK3	612	10	SMPDL3A	560

Table 15 AUC of the classification algorithms for the CML dataset

method	LAS-HDMR	LABS-HDMR	RQDA	RLDA	SVM	RF	kNN	GLM
AUC	95.65%	96.42%	96.72%	99.10%	99.18%	98.74%	97.33%	99.56%
por. (bal) var. expl.	93.57%	94.66%	94.28%	97.46%	96.53%	95.30%	95.27%	98.65%

We implement a process similar to the previous section for data analysis. We randomly select 65 points in each class for training, and the remaining points are used for testing. We again use LRT to rank genes, and the top 100 genes are used for classification. We use classifiers similar to the previous section, and iterate 100 times.

Table 15 lists the AUC of different classification rules and the proportion of the variance they explain. Note that as we use an equal number of points in each class for training, our estimates for proportion of explained variance and the proportion of balanced variance explained coincide. As the table suggests, all methods enjoy a high AUC. In this dataset linear models, such as linear probit, RLDA, and SVM enjoy the highest AUC's, but are closely followed by RQDA, LAS-HDMR, and LABS-HDMR. Linear models enjoying a high AUC suggests that for this dataset even a linear model can easily separate the two classes, and we might not need a quadratic model. The highest AUC for LAS-HDMR is obtained using RQDA with $\lambda = 0.6$, and for LABS-HDMR, RQDA with $\lambda = 0.1$ yields the highest AUC.

Again the accuracy of LAS-HDMR and LABS-HDMR might not be their most interesting property, but having the ability to explicitly say which genes and gene pairs affect the log likelihood ratio the most, which can be further used for biological verification. Table 16 lists the top 10 LAS-HDMR genes, of which many, such as DUXAP10 [31] and IGHM [32, 33, 34], are shown or suggested to be affected in CML. Histogram of top 4 genes are provided in Fig. 23. Table 16 lists the top 20 gene pairs with largest risks, i.e., gene pairs whose interactions affect the log-likelihood ratio the most. Scatter plots of several of these pairs is provided in Fig. 24. We again observe that LAS-HDMR picks gene pairs whose interactions are interesting patterns to analyze. For instance, correlation coefficient of the expression of DNTT and IGLV2-23 seems different between the two classes, and is picked by LAS-HDMR (see Fig. 24(g)).

We now study LABS-HDMR. The variant of LABS-HDMR that achieved the highest AUC used RQDA with $\lambda = 0.1$. We now use all of the data for training with this classifier to obtain the top genes, gene pairs, and their associated risk. Table 17 lists the top 10 LABS-HDMR genes and their risks, and Fig. 25 provides expression histograms across the two classes for some of these genes. It is again no surprise to us that top genes are extremely differentially expressed and have been shown to be affected in CML. For example, COL9A2 [35] and CTSB [36, 37] genes are shown to be involved in CML.

Table 17 lists the top 20 gene pairs whose interactions had the largest risks. Although these gene pairs have rather large risks due to their interactions, they

Table 16 Top CML gene pairs used for classification by LAS-HDMR

Rank	Gene 1	Gene 2	Risk Type	Risk Amount
1	IGLV2-23	DUXAP10	increasing	17.6712
2	IGK, IGKC	DUXAP10	increasing	16.9648
3	IGLV2-23	SMPDL3A	increasing	16.809
4	IGKV4-1	DUXAP10	increasing	16.7693
5	IGK, IGKC	SMPDL3A	increasing	16.6523
6	IGKV4-1	SMPDL3A	increasing	16.4429
7	KIF21A	IGLV2-23	increasing	15.6573
8	IGK, IGKC	KIF21A	increasing	15.5903
9	LY9	IGLV2-23	increasing	15.5559
10	IGH(A1-D-G1,3-M,V1,3,4), LOC100126583	DUXAP10	increasing	15.5457
11	IGL,	DUXAP10	increasing	15.5086
12	ITPR3	IGLV2-23	increasing	15.4596
13	ITPR3	DUXAP10	increasing	15.4441
14	IGK, IGKC	LY9	increasing	15.3819
15	DNTT	IGLV2-23	increasing	15.3116
16	NMU	ITPR3	increasing	15.2687
17	IGL,	SMPDL3A	increasing	15.1065
18	IGKV4-1	LY9	increasing	15.0803
19	IGK, IGKC	ITPR3	increasing	15.074
20	IGHM LOC100133862	DUXAP10	increasing	15.0578

Table 17 Top CML genes used for classification by LABS-HDMR

Rank	Gene	Risk Amount	Rank	Gene	Risk Amount
1	CCDC109B	899.4738	6	ABL1	755.8205
2	LOC91316	802.3985	7	IRF8	738.7324
3	COL9A2	794.5245	8	IGLL3P	736.5587
4	CTSB	781.2246	9	IGHM	724.3461
5	LTC4S	767.4666	10	IRF4	691.3449

Table 18 Top CML gene pairs used for classification by LAS-HDMR

Rank	Gene 1	Gene 2	Risk Type	Risk Amount
1	PTK2	SMPDL3A	increasing	22.7551
2	AQP3	SMPDL3A	increasing	22.7551
3	SMPDL3A	IGJ	increasing	21.6855
4	BLNK	SMPDL3A	increasing	21.6855
5	IGLV2-23	SMPDL3A	increasing	20.3118
6	IGK@ IGKC	SMPDL3A	increasing	20.3118
7	SERPINF1	SMPDL3A	increasing	19.536
8	IGHM	SMPDL3A	increasing	19.536
9	IGKV4-1	SMPDL3A	increasing	19.5037
10	NMU	LY9	increasing	19.5037
11	DUXAP10	IGJ	increasing	19.3916
12	IGF2BP2	SMPDL3A	increasing	19.3916
13	ANKH	SMPDL3A	increasing	19.382
14	IGHM	DUXAP10	increasing	19.382
15	PRKCH	IGF2BP2	increasing	18.8683
16	GCNT2	SMPDL3A	increasing	18.8683
17	LY9	SMPDL3A	increasing	18.4606
18	LY9	DUXAP10	increasing	18.4606
19	LTC4S	PTK2	increasing	18.1344
20	KIF21A	IGJ	increasing	18.1344

are still much smaller than the risk of individual genes. Figure 26 provides scatter plots of several of the gene pairs with largest risks. Note a general pattern observed across the gene pairs is that (a) two genes are both down-regulated across CML patients, and (b) the expression values have larger variances among CML patients compared with healthy people.

Table 19 lists the number of significant pairwise interactions detected by each test. For each gene we again only use the probe ranking highest by LRT, so that probes mapping to the same genes do not disrupt the analysis, and perform outlier detection. This way we get 21049 different genes, which give us 221519676 different pairwise dependence tests. Bounding FDR by 5% we see that 5820338 gene pairs, about 2.63% of all tests, are significant. Table 19 lists the top 25 gene pairs and their adjusted p-values. Figure 28 provides scatter plots of several gene pairs.

Table 19 Top 25 gene pairs of the CML dataset

Rank	gene 1	gene 2	adj P-value (col $\times 10^{-27}$)
1	IGLV1-44 LOC100290481	IGHG1 IGHG2 IGHM IGHV4-31	$< 10^{-4}$
2	IGLV1-44 LOC100290481	IGHA1 IGHA2 LOC100126583	$< 10^{-4}$
3	IGLV1-44 LOC100290481	IGLL3P	$< 10^{-4}$
4	IGLC7 IGLV1-44 LOC100290481	IGHA1 IGHA2 LOC100126583	$< 10^{-4}$
5	IGHG1 IGHG2 IGHM IGHV4-31	IGLC7 IGLV1-44	$< 10^{-4}$
6	IGLV1-44 LOC100290481	LOC91316	$< 10^{-4}$
7	IGHG1 IGHG2 IGHM IGHV4-31	IGK@ IGKC IGKV1-5	$< 10^{-4}$
8	IGLC7 IGLV1-44	IGHA1 IGHA2 LOC100126583	$< 10^{-4}$
9	IGHG1 IGHG2 IGHM IGHV4-31	IGLC7 IGLV1-44 LOC100290481	$< 10^{-4}$
10	LOC91316	IGLC7 IGLV1-44	$< 10^{-4}$
11	IGLV1-44 LOC100290481	IGK@ IGKC IGKV1-5	$< 10^{-4}$
12	IGLC7 IGLV1-44	IGLL5 IGLV2-11	$< 10^{-4}$
13	IGLC7 IGLV1-44	IGLL3P	0.0006
14	IGK@ IGKC IGKV1-5	IGHA1 IGHA2 LOC100126583	0.0008
15	IGLV1-44 LOC100290481	IGLL5 IGLV2-11	0.0061
16	LOC91316	IGLC7 IGLV1-44 LOC100290481	0.0152
17	IGK@ IGKC	IGLC7 IGLV1-44	0.0199
18	IGLC7 IGLV1-44	IGLV2-23	0.0199
19	IGLC7 IGLV1-44	IGKC IGKV1-5 IGKV1D-8 LOC652493 LOC652694	0.0255
20	IGLC7 IGLV1-44 LOC100290481	IGLL3P	0.0308
21	IGHG1 IGHG2 IGHM IGHV4-31	IGHA1 IGHA2 LOC100126583	0.0582
22	IGLV1-44 LOC100290481	IGLC7 IGLV1-44	0.0667
23	IGJ	IGLC7 IGLV1-44	0.082
24	IGK@ IGKC IGKV1-5	IGLC7 IGLV1-44	0.137
25	IGKC	IGLC7 IGLV1-44	0.2103

2.3.1 Network Analysis Results

Here we provide further details regarding the IPA results. We again selected the top 200 genes based on their $p(g)$'s, and consider the graph of selected genes and significant pairwise interactions where edge eights are $-\log(P_{MTM}(g, g'))$. We then use the graph community detection algorithm of [23] to cluster genes to communities, giving us 7 disjoint communities. Figure 29 plots the largest detected community, containing 146 genes. We would to emphasize this community of dysregulated co-expressions is highly connected, containing 6094 nodes. We then use these genes IPA, only using experimentally validated and strong interactions. IPA detects 9 networks, where the top network is provided in Fig. 30, containing 14 genes of our inputted list, and corresponds to cell cycle, cellular maintenance and function, and cellular development processes. We again use log fold changes to label which genes are over/under-expressed. Note many of the genes are connected directly or with a single gene in between. Finally, Fig. 31 provides the top IPA pathways corresponding to this gene cluster.

3 Degenerate Cases for Pairwise Gaussian Feature Interactions

Here we study the degenerate case of pairwise feature interactions for Gaussian features, i.e., the case where two features have the same covariances in both classes, but one of them has similar means in both classes.

Consider the feature pair f_i, f_j, and assume they are jointly Gaussian in each class. Suppose f_i, f_j are correlated, and have the same covariance matrix in both classes, hereafter denoted by Σ^{f_i,f_j}. Note if both f_i and f_j have similar means in both classes, then they have the same distribution in both classes, and cannot carry information about class labels. Additionally, the case where they both have different means between the two classes is studied in the main paper. Here we consider the case where one feature has similar means in both classes while the other feature has different means. Without loss of generality assume $\mu_1^{f_i} \neq \mu_0^{f_i}$ and $\mu_1^{f_j} = \mu_0^{f_j}$. We already know that the log likelihood ratio is found in the LDA process; however, we now study how the fact that $\mu_1^{f_j} = \mu_0^{f_j}$ affects the HDMR expansion.

Note that since we have assumed covariances are similar in both classes, f_j has similar variances in both classes. Additionally, we have assumed that $\mu_1^{f_j} = \mu_0^{f_j}$. Therefore, $E(L(X)|X_j) = 0$, i.e., given only the value of X_j, the log likelihood ratio is a constant. This results in $S(X_j) = 0$, and hence X_j does not enter the first order HDMR expansion. Although we know that we only need linear terms of X_j to find the log likelihood ratio, we need to look at second order expansion. In the second order expansion, although the second degree terms cancel out, the first order terms remain, and give us the desired result. Therefore, in this special case, although we have a linear classifier, we need to consider the second order expansion to properly include f_j in the classification rule. This suggests that under this special case, there is a feature interaction relation between f_i and f_j, which should be considered. Therefore, we need the following adjustment to the null of MTM:

$$
\begin{aligned}
&H_0 : \rho_0^{f_i,f_j} = \rho_1^{f_i,f_j} = 0 \ \text{ or } \ \Big[\Big(\Sigma_0^{f_i,f_j} = \Sigma_1^{f_i,f_j}\Big) \text{ and} \\
&\Big(\Big((\mu_1^{f_i} \neq \mu_0^{f_i})\text{and}(\mu_1^{f_j} \neq \mu_0^{f_j})\Big) \text{ or } \Big((\mu_1^{f_i} = \mu_0^{f_i})\text{and}(\mu_1^{f_j} = \mu_0^{f_j})\Big)\Big)\Big] \\
&v.s. \qquad H_1 : \text{otherwise.}
\end{aligned}
$$

Identifying this null is indeed very difficult, and here we aim to properly approximate its distribution. Note have already handled the cases for $\rho_0^{f_i,f_j} = \rho_1^{f_i,f_j} = 0$ and $\Sigma_0^{f_i,f_j} = \Sigma_1^{f_i,f_j}$ in the main paper. Now we aim to assign a p-value to the event

$$
\Big((\mu_1^{f_i} \neq \mu_0^{f_i})\text{and}(\mu_1^{f_j} \neq \mu_0^{f_j})\Big) \text{ or } \Big((\mu_1^{f_i} = \mu_0^{f_i})\text{and}(\mu_1^{f_j} = \mu_0^{f_j})\Big).
$$

First consider the case that $(\mu_1^{f_i} = \mu_0^{f_i})\text{and}(\mu_1^{f_j} = \mu_0^{f_j})$. Let

$$
d^f = \frac{\hat{\mu}_1^f - \hat{\mu}_0^f}{\sqrt{\hat{\sigma}_1^f/(n_1-1) + \hat{\sigma}_0^f/(n_0-1)}}, \tag{3}
$$

where $\hat{\mu}_y^f$ and $\hat{\sigma}_y^f$ denote sample mean and sample variance of feature f in class y, respectively. Note that we can identify $d^{f_i,f_j} = [d^{f_i},\ d^{f_j}]$ as a point in $\mathbf{R}^2$. In the event that $(\mu_1^{f_i} = \mu_0^{f_i})\text{and}(\mu_1^{f_j} = \mu_0^{f_j})$, we estimate the distribution of $||d^{f_i,f_j}||_2$ with a χ^2 distribution. We also have that

$$
\begin{aligned}
\theta^{f_i,f_j} &= \max\{|\arctan \mu_1^{f_i}/\mu_0^{f_i}|, \pi/2 - |\arctan \mu_1^{f_i}/\mu_0^{f_i}|\} \\
&\sim uniform([0,\pi/4]).
\end{aligned}
$$

We assume that when $(\mu_1^{f_i} \neq \mu_0^{f_i})\text{and}(\mu_1^{f_j} \neq \mu_0^{f_j})$, then $||d^{f_i,f_j}||$ is large, getting a very small p-value for the χ^2 test, and θ^{f_i,f_j} is still uniformly distributed on the $[0,\pi/4]$ interval. Therefore, we use the maximum of the $||d^{f_i,f_j}||$ and θ^{f_i,f_j} p-values, as the final p-value statistic. We then take the minimum between the p-values of means both equal or unequal, and proceed as before. Although such approach may actually underestimate the tail probability of the null, i.e., the p-value, we observed that examples of these degenerate cases are indeed very rare in the analyzed datasets, and such approach should not heavily overwhelm the analysis. We hereafter call this test MTM complete (MTM-C).

We now study how the use of MTM-C instead of MTM affects the real expression data analyses. In the breast cancer dataset MTM-C did not output any new identified patterns. Figure 32(a) provides the scatter plot of the feature pair with smallest $P_p + P_m$, where P_p is the p-value of the Pearson correlation part, and P_m is the p-value of the mean comparison part. The minimum raw value was 1.5269×10^{-5}, which is indeed not significant bounding FDR by 0.05 using BH. Note this pair is significant bounding FDR by 0.05 using MTM and MTM-C due to differences in covariance.

In the CML dataset we again observe that $\min P_p + P_m = 7.8 \times 10^{-5}$, and the scatter plot of the feature pair obtaining this p-value is provided in Fig. 32(b). We again observe that this p-value is not significant after FDR correction. however, this might be an interesting feature pair interaction and a missed discovery. Note that FAM169A alone only explains 73.47% of the balanced variance, but the feature pair PRPF4 and FAM169A can explain 78.77% of the balanced variance using a linear classification rule. We observed this feature pair is significant under MTM due to differences in covariance.

In the lung cancer dataset, $\min P_p + P_m = 1.7242 \times 10^{-5}$, not significant when bounding FDR by 5%, and the scatter plot of the feature pair obtaining this p-value is provided in Fig. 32(c). We observed these two features explain 26.17% of the balanced variance, while the NFIB gene alone can only explain 21.46% of the variance. Hence, this pair might be a missed discovery. As the scatter plot suggests, under-expression of NFIB and over-expression of C1orf79 might be an indicator of high chance of relapse or death. Note this pair is not significant under MTM either.

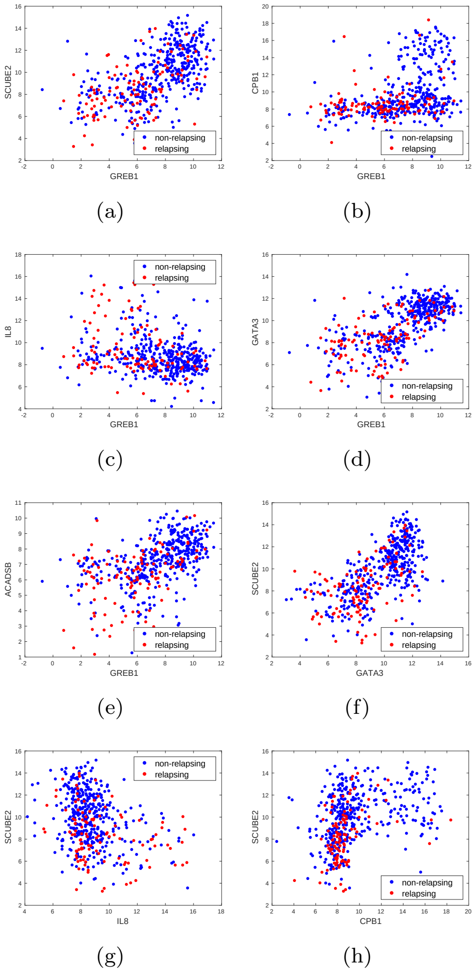

Figure 5 Scatter plots of LAS-HDMR gene pairs with large risks values of the breast cancer dataset: (a) Lower expression of both GREB1 and SCUBE2 is an indicator of high relapse risk. (b) Over expression of GREB1 and CPB1 is an indicator of low risk, over expression of GREB1 and low expression of CPB1 is an indicator of medium risk, and low expression of both is an indicator of high risk. (c) Over expression GREB1 and low expression of IL8 is an indicator of low risk. (d) Over expression of both GREB1 and GATA3 is an indicator of low risk. (e) Over expression of both GREB1 and ACADSb is an indicator of low risk. (f) Over expression of both SCUBE2 and GATA3 is an indicator of low risk. (g) Over expression of IL8 and low expression of SCUBE2 is an indicator of high risk. (h) High expression of CPB1 and SCUBE2 is an indicator of low risk.

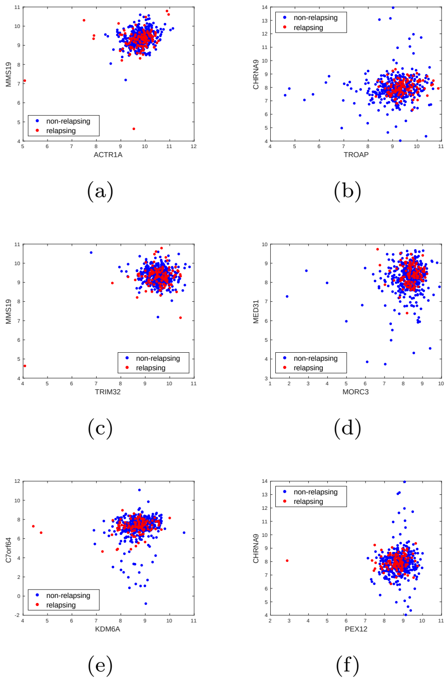

Figure 6 Scatter plots of several LAS-HDMR gene pairs with small associated risks comparing relapsing and non-relapsing breast cancer patients. These interactions are not used by LAS-HDMR as the information the gene pairs provide seem to be already encoded in the first order HDMR expansion.

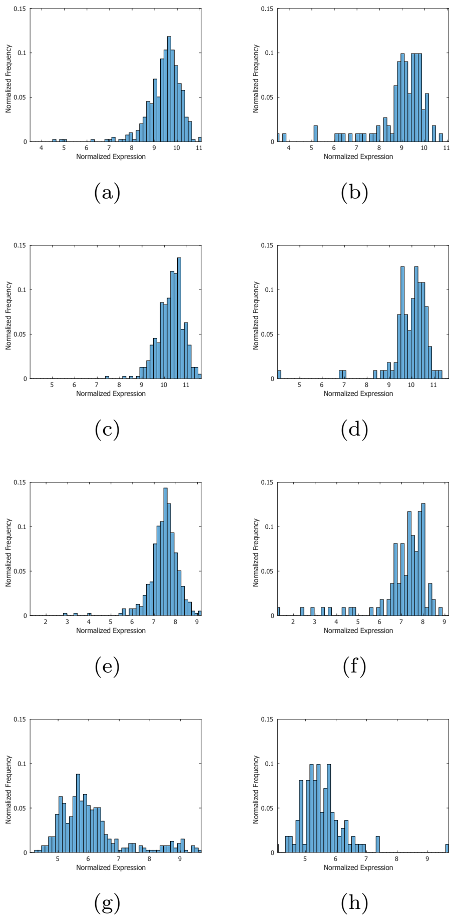

Figure 7 Histogram of breast cancer genes ranking high by LABS-HDMR: (a) non-relapsing GMPR2, (b) relapsing GMPR2, (c) non-relapsing TOX4, (d) relapsing TOX4, (e) non-relapsing IGBP1, and (f) relapsing IGBP1, suggesting GMPR2, TOX4, and IGBP1 have lower expression in a subpopulation of relapsing patients, and (g) non-relapsing ACTR1A, and (h) relapsing ACTR1A, suggesting ACTR1A has higher expression in a subpopulation of relapsing patients.

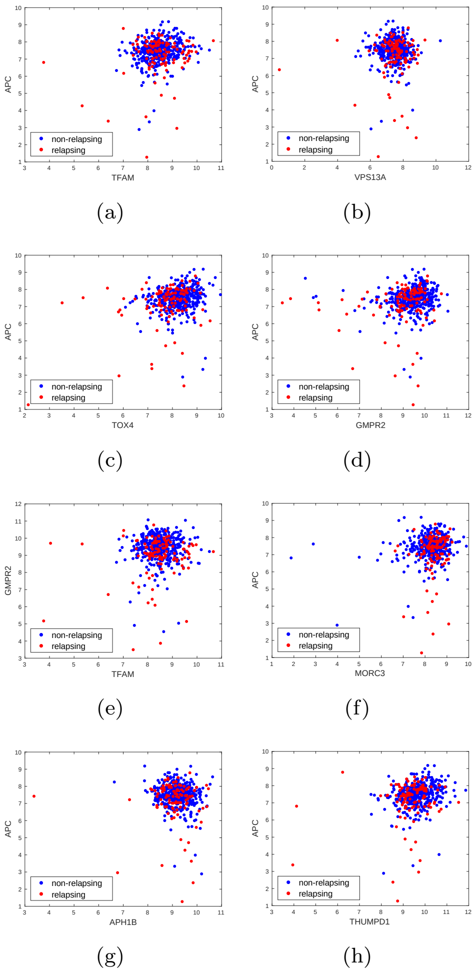

Figure 8 Scatter plots of LABS-HDMR gene pairs with large risks values of the breast cancer dataset. In contrast to LAS-HDMR, LABS-HDMR assigns large risks to gene pairs that are heavily affected for small subpopulations, due to the built in averaging property, merging the less prevalent patterns to arrive at a normalized risk for each block.

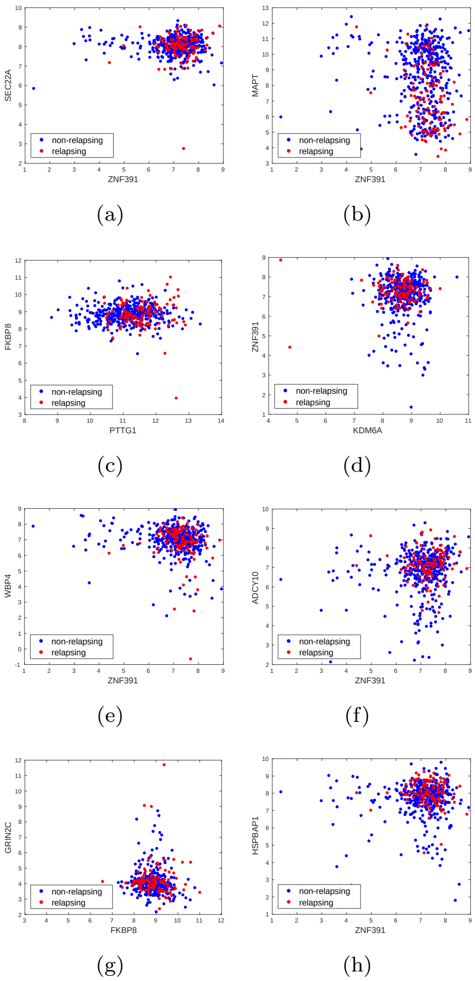

Figure 9 Scatter plots of several LABS-HDMR gene pairs with small associated risks by LABS-HDMR, i.e., no significant interactions, comparing relapsing and non-relapsing breast cancer patients.

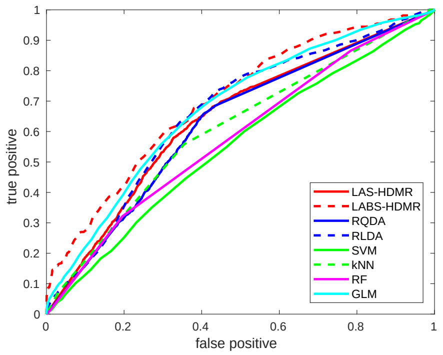

Figure 10 ROC curve of different classifiers for the breast cancer dataset.

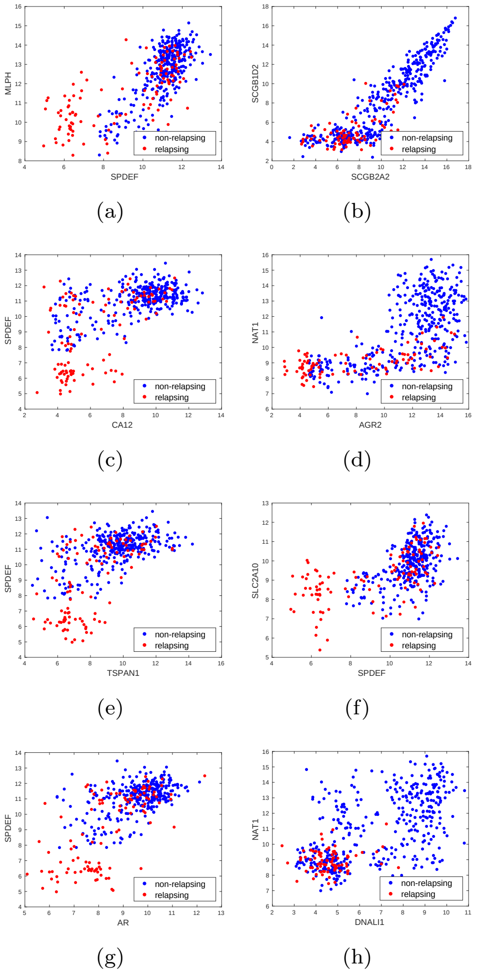

Figure 11 Scatter plots of gene pairs with significant interactions for the breast cancer dataset by MTM. Over expression of (a) SPDEF and MLPH, (b) SCGB2A2 and SCGB1D2, (c) CA12 and SPDEF, (d) AGR2 and NAT1, (e) TSPAN1 and SPDEF, (f) SPDEF and SLC2A10, and (g) SPDEF and AR are all indicators of low risk. (h) Low expression of both NAT1 and DNALI1 is an indicator of high relapse risk.

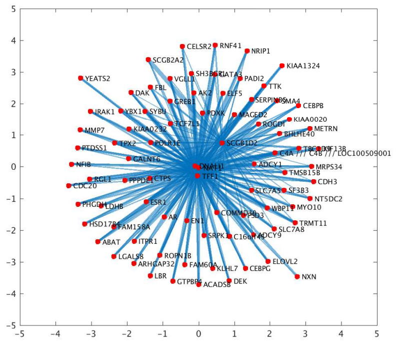

Figure 12 The largest MTM gene cluster of the breast cancer dataset. SCGB1D2, TFF1, NAT1, and DNALI1 seems heavily connected to many other genes.

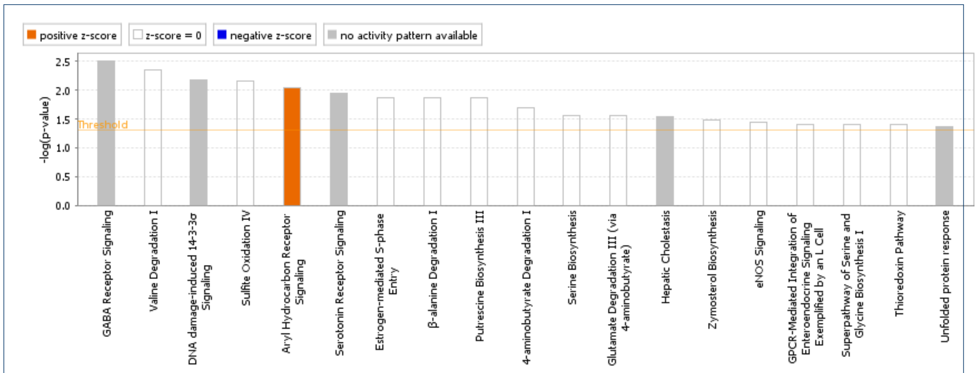

Figure 13 Top IPA canonical pathways of the largest MTM gene cluster of the breast cancer dataset.

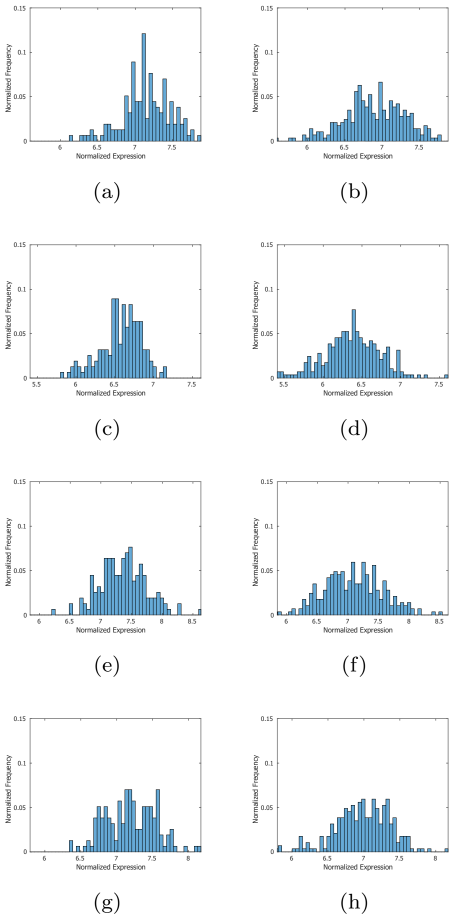

Figure 14 Histogram of lung cancer genes ranking high by LAS-HDMR: (a) survived BPTF, (b) dead or relapsed BPTF, (c) survived SEC63, (d) dead or relapsed SEC63, (e) survived UBXN4, (f) dead or relapsed UBXN4, (g) survived SRSF2IP, and (h) dead or relapsed SRSF2IP. All histograms presented here suggest their associated genes have lower expressions among high risk (relapsed or dead) patients.

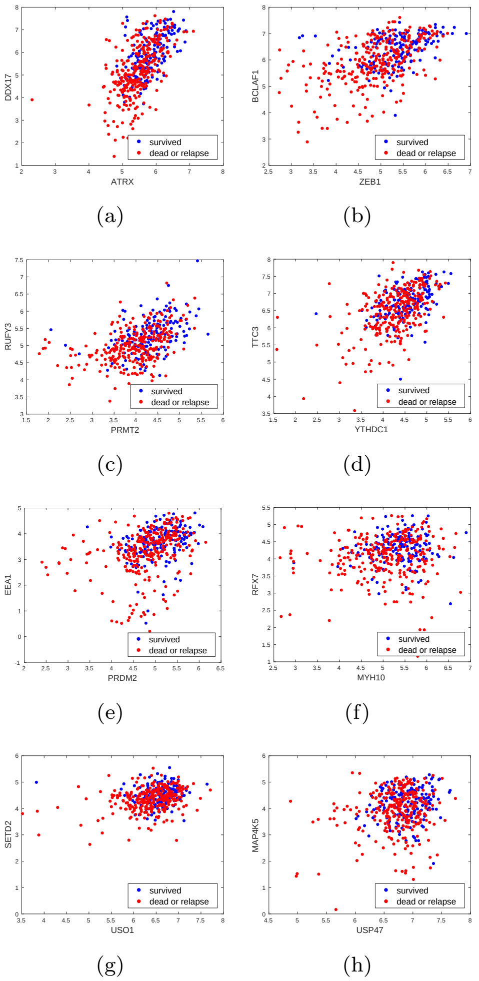

Figure 15 Scatter plots of several LAS-HDMR gene pairs with large risks values of the lung cancer dataset: (a) low expression of ATRX and DDX17, (b) low expression of ZEB1 and BCLAF1, low expression of both (c) PRMT2 and RUFY3, or (d) YTHDC1 and TTC3, (e) low expression of PRDM2 or EEA1, (f) low expression of MYH10 and high expression of RFX7, and (h) low expression of USP47 or MAP4K5 are all indicators of high risk. (g) Low expression of USO1 is an indicator of high risk. USO1 and SETD2 are significantly positively correlated among high risk patients and USO1 has larger variance among high risk patients. Slight lower expression of SETD2, when accompanied with low expression of USO1, is a strong indicator of high risk.

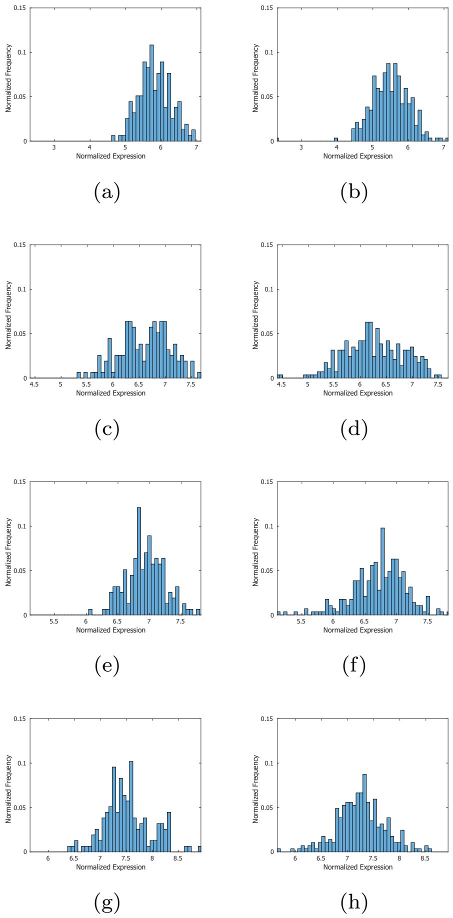

Figure 16 Histogram of lung cancer genes ranking high by LAS-HDMR: (a) survived ATRX, (b) dead or relapsed ATRX, (c) survived SON, (d) dead or relapsed SON, (e) survived KIAA1033, (f) dead or relapsed KIAA1033, (g) survived LUC7L3, and (h) dead or relapsed LUC7L3. Again all these genes seem to have lower expressions among high risk patients.

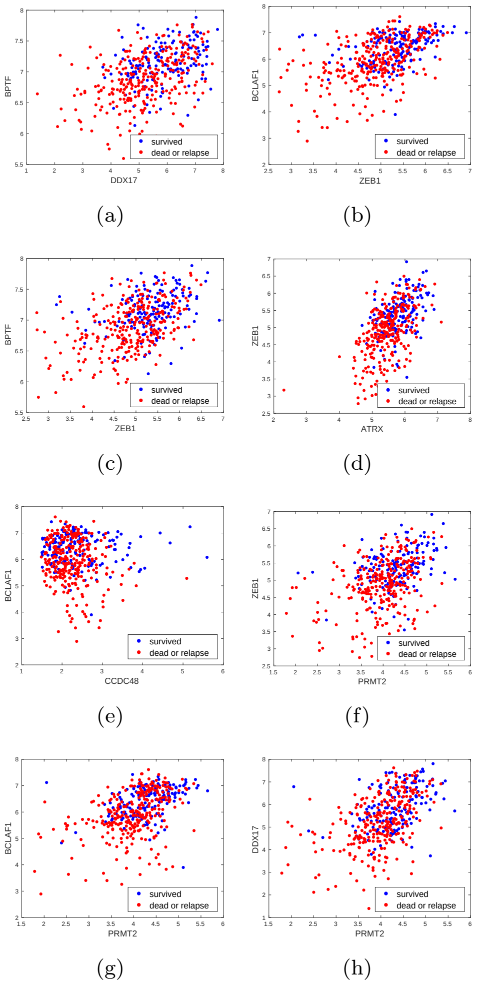

Figure 17 Scatter plots of several LABS-HDMR gene pairs with large risks values of the lung cancer dataset. Lower expressions of (a) DDX17 and BPTF, (b) ZEB1 and BCLAF1, (c) ZEB1 and BPTF, (d) ATRX and ZEB1, (f) PRMT2 and ZEB1, (g) PRMT2 or BCLAF1, and (h) PRMT2 and DDX17 seem to be indicators of high risk patients. (e) Over expression of CCDC48 and BCLAF1 seems to be an indicator of low risk, low expression of both CCDC48 and BCLAF1 is an indicator of low risk, and low expression of CCDC48 and high expression of BCLAF1 is best described by a medium risk.

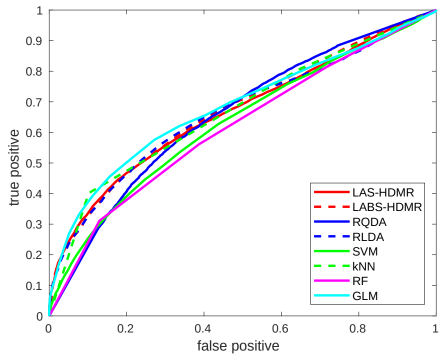

Figure 18 ROC curve of different classifiers for the lung cancer dataset.

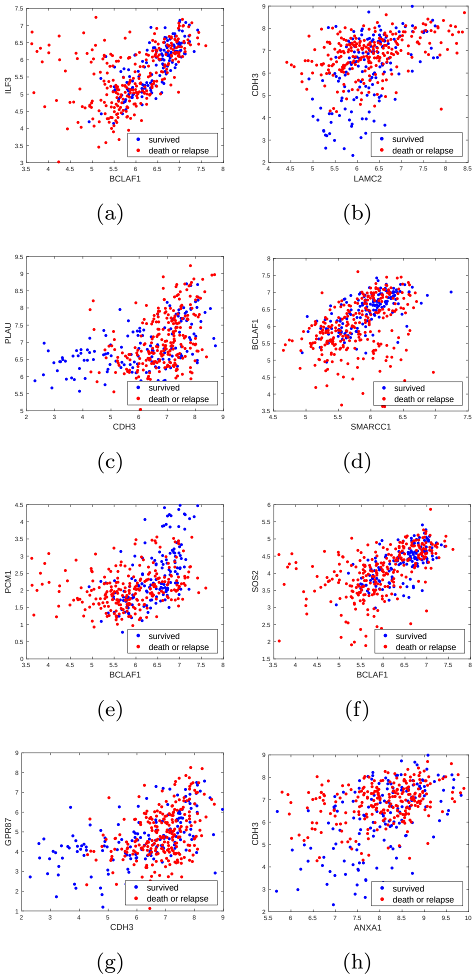

Figure 19 Scatter plots of gene pairs with significant interactions for the lung cancer dataset. (a) A subpopulation of high risk patients have low BCLAF1 expression and high ILF3 expression. (b) Over expression of both LAMC2 and CDH3 is an indicator of high risk, low expression of both is an indicator of low risk, and over expression of CDH3 and low expression of LAMC2 is an indicator of medium risk. (c) Over expression of CDH3 and PLAU is an indicator of high risk, low expression of both is an indicator of low risk, and over expression of CDH3 and low expression of PLAU is an indicator of medium risk. (d) For a subpopulation of high risk patients SMARCC1 has high and BCLAF1 has low expression. (e) Over expression of both BCLAF1 and PCM1 is an indicator of low risk, low expression of both is an indicator of high risk, and high expression of BCLAF and low expression of PCM1 is an indicator of medium risk. (f) Low expression of BCLAF1 or SOS2 is an indicator of high risk. (g) Low expression of both CDH3 and GPR87 is an indicator of low risk. (h) Over expression of both ANXA1 and CDH3 is and indicator of high risk, low expression of both is an indicator of low risk, and over expression of CDH3 accompanied with low expression of ANXA1 is an indicator of high risk.

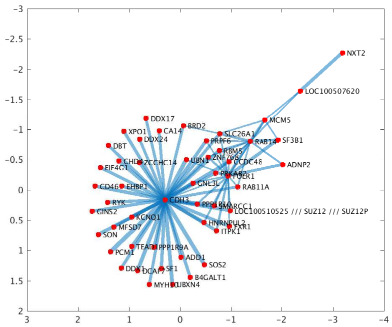

Figure 20 The largest MTM gene cluster of the lung cancer dataset. CDH3, FOER1, PRKAB2, and RAB14 seem heavily connected to other genes.

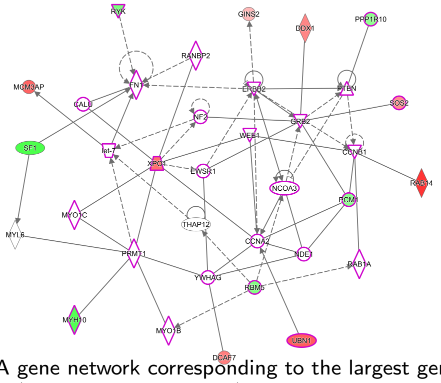

Figure 21 The largest IPA gene network corresponding to the largest gene cluster of MTM for the lung cancer dataset. Green/Red denote decreased/increased expression among high risk patients compared with low risk patients. Solid/Dashed lines denote direct/indirect interactions. Genes highlighted with magenta borders are associated with cell cycle, cellular assembly and organization, and cellular function and maintenance. Many dysregulated gene pairs seem to be affected via a mediating gene. Note the effect of each individual gene is more pronounced than the breast cancer dataset.

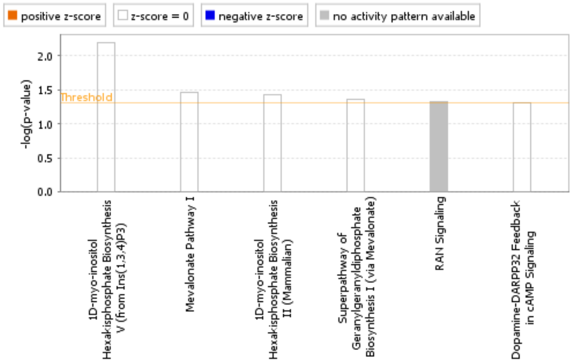

Figure 22 Top IPA canonical pathways of the largest MTM gene cluster of the lung cancer dataset.

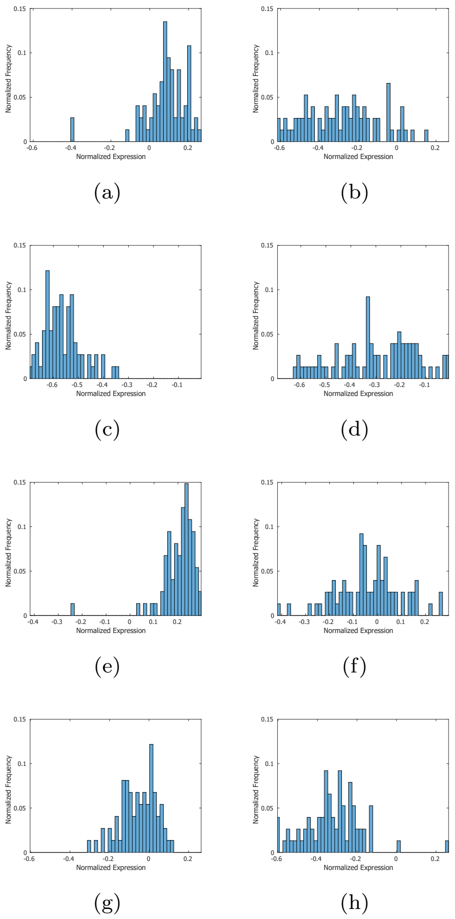

Figure 23 Histogram of CML genes ranking high by LAS-HDMR: (a) healthy IGLV2-23, (b) CML IGLV2-23, (c) healthy DUXAP10, (d) CML DUXAP10, (e) healthy IGHM, (f) CML IGHM, (g) healthy BLNK, and (h) CML BLNK. These figures suggest IGLV2-23, DUXAP10, IGHM, and BLNK have lower, higher, lower, and lower expressions among CML patients compared with healthy people, respectively.

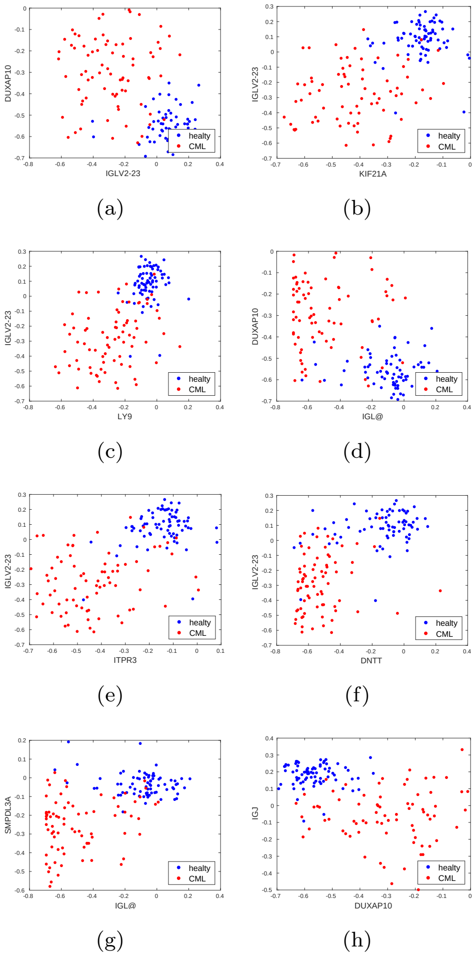

Figure 24 Scatter plots of several LAS-HDMR gene pairs with large risks values of the CML dataset. All figures suggest the gene dependencies provide information about their possible effect in CML. In particular, in part (d) we see IGL has lower expression than DUXAP10 among all healthy people while has higher expression among almost all CML patients. Similar patterns exist among gene pairs in parts (f) and (g) as well.

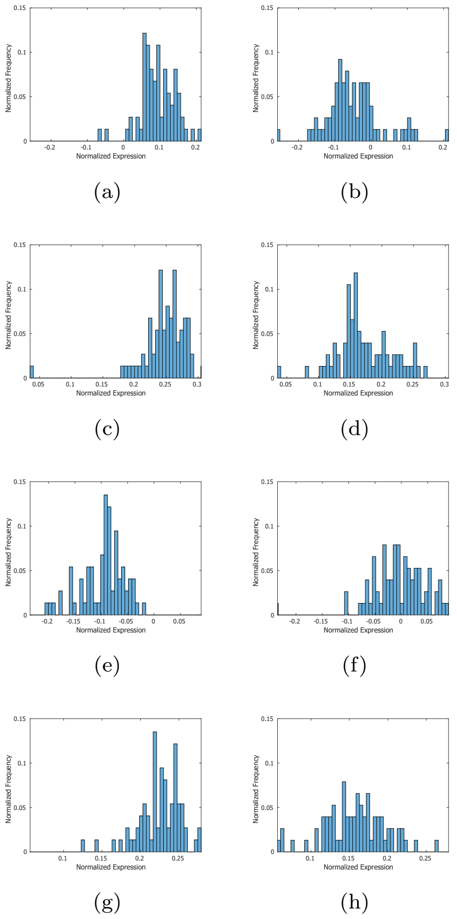

Figure 25 Histogram of CML genes ranking high by LABS-HDMR: (a) healthy CCDC109B, (b) CML CCDC109B, (c) healthy LOC91316, (d) CML LOC91316, (e) healthy COL9A2, (f) CML COL9A2, (g) healthy CTSB, and (h) CML CTSB. CCDC109B, LOC91316, COL9A2, and CTSB seem to have lower, lower, higher, and lower expressions among CML patients, respectively.

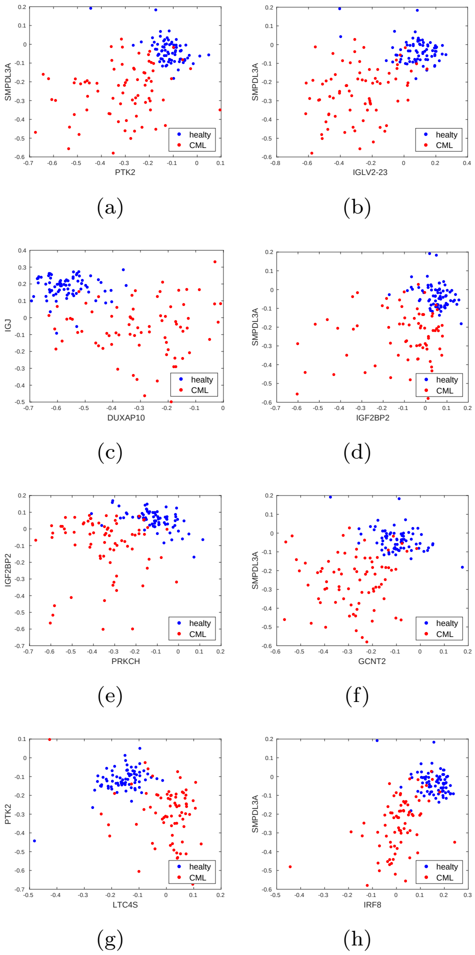

Figure 26 Scatter plots of several LABS-HDMR gene pairs with large risks values of the CML dataset. Again the interactions between gene pairs provides information not present accounting for each one separately, for instance, in part (g) we see LTC4S has higher expression than PTK2 among CML patients while has higher expression among healthy people.

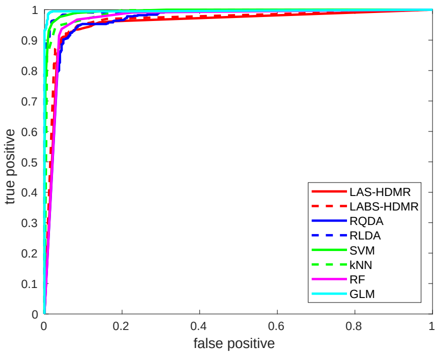

Figure 27 ROC curve of different classifiers for the CML dataset.

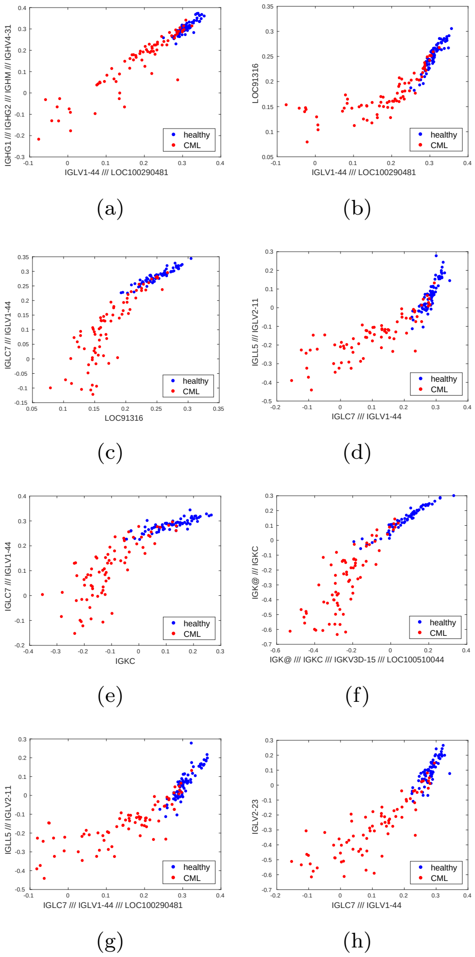

Figure 28 Scatter plots of gene pairs with significant interactions for the CML dataset. This features suggest that expression levels seem to have much larger variances among CML patients and have different correlation coefficients compared with healthy people.

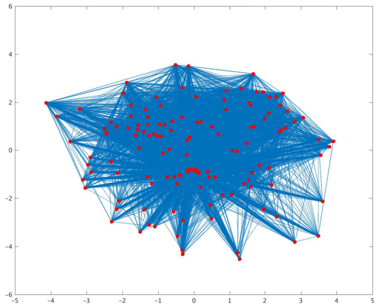

Figure 29 The largest MTM gene cluster of the CML dataset. Due to the large number of nodes the gene names are not shown.

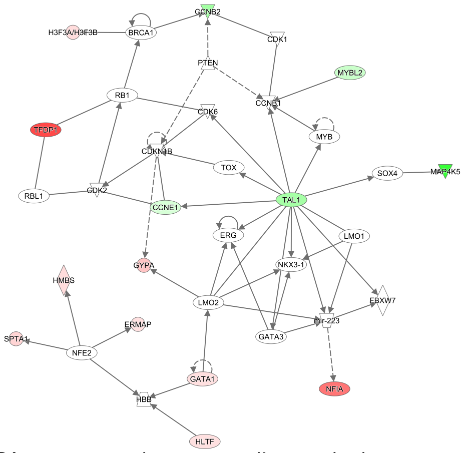

Figure 30 The largest IPA gene network corresponding to the largest gene cluster of MTM for the CML dataset. The genes at the bottom left portion of the figure seem to be a small subnetwork, and TAL1 and NKX3-1 seem to the be centers of another subnetwork. These subnetworks are connected through LMO2, which is suggested to be necessary for hematopoiesis [38].

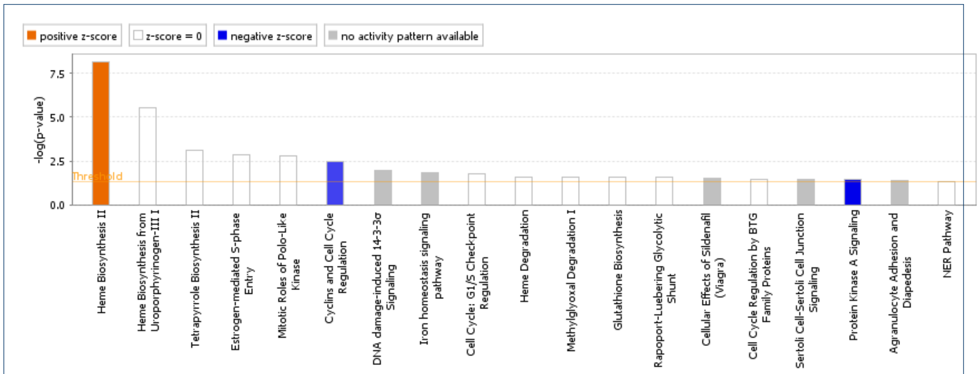

Figure 31 Top IPA canonical pathways of the largest MTM gene cluster of the CML dataset.

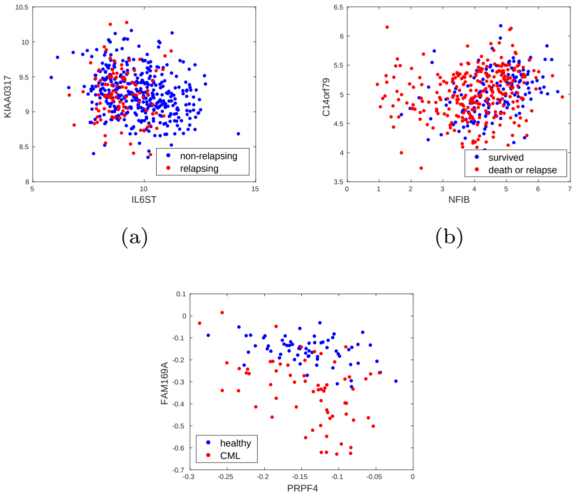

(a) (b) (c)

Figure 32 Gene pairs with largest interactions where one gene tends to have equal means in both classes for the (a) breast cancer, (b) lung cancer, and (c) CML datasets. (a) KIAA0317 seems to have similar distributions between relapsing and not relapsing patients, while its correlation coefficient with IL6ST differs between he two classes. (b) C14orf79 seems to have similar distributions between the two lung cancer patients while NFIB has lower expression among a subpopulation of high risk patients. he positive correlation coefficient between NFIB and C14orf79 helps to better detect few high risk points with NFIB expression between 2 and 4. (c) PRPF4 seems to follow similar distributions among healthy people and CML patients while FAM169A has lower expression among CML patients; however, the correlation between PRPF4 and FAM169A helps separating points with FAM169A expression between -0.2 and -0.35.

Author details
[1]Department of Electrical and Computer Engineering, The Ohio State University, 205 Dreese laboratories, 2015 Neil Ave., 43210 Columbus, USA. [2]Department of Mathematics, The Ohio State University, 100 Math Tower, 31 West 18th Ave., 43210 Columbus, USA. [3]Department of Biomedical Informatics, The Ohio State University, 1585 Neil Ave, 43210 Columbus, USA. [4]College of Public Health, 250 Cunz Hall, 1841 Neil Ave., 43210 Columbus, USA.

References

1. Hajian-Tilaki, K.: Sample size estimation in diagnostic test studies of biomedical informatics. Journal of Biomedical Informatics **48**, 193–204 (2014)
2. Obuchowski, N.A., McClish, D.K.: Sample size determination for diagnostic accuracy studies involving binormal ROC curve indices. Statistics in medicine **16**(13), 1529–1542 (1997)
3. Hanley, J.A., McNeil, B.J.: A method of comparing the areas under receiver operating characteristic curves derived from the same cases. Radiology **148**(3), 839–843 (1983)
4. Hwang, D., Schmitt, W.A., Stephanopoulos, G., Stephanopoulos, G.: Determination of minimum sample size and discriminatory expression patterns in microarray data. Bioinformatics **18**(9), 1184–1193 (2002)
5. Saccenti, E., Timmerman, M.E.: Approaches to sample size determination for multivariate data: Applications to PCA and PLS-DA of omics data. Journal of proteome research **15**(8), 2379–2393 (2016)
6. Xing, P., Chen, Y., Gao, J., Bai, L., Yuan, Z.: A fast approach to detect gene–gene synergy. Scientific reports **7**(1), 1–8 (2017)
7. Itoh, M., Iwamoto, T., *et al.*: Estrogen receptor (ER) mRNA expression and molecular subtype distribution in ER-negative/progesterone receptor-positive breast cancers. Breast Cancer Research and Treatment **143**(2), 403–409 (2014)
8. Hatzis, C., Pusztai, L., *et al.*: A genomic predictor of response and survival following taxane-anthracycline chemotherapy for invasive breast cancer. JAMA **305**(18), 1873–1881 (2011)
9. Edgar, R., Domrachev, M., *et al.*: Gene expression omnibus: NCBI gene expression and hybridization array data repository. Nucleic Acids Res. **30**(1), 207–210 (2002)
10. Pearson, E.S., Neyman, J.: On the problem of two samples. In: Neyman, J., Pearson, E.S. (eds.) Joint Statistical Papers (1967), pp. 99–115 (1930)
11. Zhang, L., Xu, X., Chen, G.: The exact likelihood ratio test for equality of two normal populations. The American Statistician **66**(3), 180–184 (2012)
12. Finak, G., Bertos, N., *et al.*: Stromal gene expression predicts clinical outcome in breast cancer. Nature Medicine **14**(5), 518–527 (2008)
13. Korkaya, H., Kim, G.-i., Davis, A., Malik, F., Henry, N.L., Ithimakin, S., Quraishi, A.A., Tawakkol, N., D'Angelo, R., Paulson, A.K., Chung, S., Luther, T., Paholak, H.J., Liu, S., Hassan, K.A., Zen, Q., Clouthier, S.G., Wicha, M.S.: Activation of an il6 inflammatory loop mediates trastuzumab resistance in HER2+ breast cancer by expanding the cancer stem cell population. Molecular Cell **47**(4), 570–584 (2012)
14. Ginestier, C., Liu, S., Diebel, M.E., Korkaya, H., Luo, M., Brown, M., Wicinski, J., Cabaud, O., Charafe-Jauffret, E., Birnbaum, D., Guan, J.-L., Gabriela, D., Wicha, M.S.: CXCR1 blockade selectively targets human breast cancer stem cells in vitro and in xenografts. The Journal of Clinical Investigation **120**(2), 485–497 (2010)
15. Rae, J.M., Johnson, M.D., *et al.*: GREB1 is a critical regulator of hormone dependent breast cancer growth. Breast Cancer Research and Treatment **92**(2), 141–149 (2005)
16. Ghosh, M.G., Thompson, D.A., Weigel, R.J.: PDZK1 and GREB1 are estrogen-regulated genes expressed in hormone-responsive breast cancer. Cancer Research **60**(22), 6367–6375 (2000)
17. Sun, J., Nawaz, Z., Slingerland, J.M.: Long-range activation of GREB1 by estrogen receptor via three distal consensus estrogen-responsive elements in breast cancer cells. Molecular Endocrinology **21**(11), 2651–2662 (2007)
18. Baker, B.G., Ball, G..R., Rakha, E.A., Nolan, C.C., Caldas, C., Ellis, I.O., Green, A.R.: Lack of expression of the proteins GMPR2 and PPARα are associated with the basal phenotype and patient outcome in breast cancer. Breast Cancer Research and Treatment **137**(1), 127–137 (2013)
19. Park, N.I., Rogan, P.K., *et al.*: Structural and genic characterization of stable genomic regions in breast cancer: relevance to chemotherapy. Molecular Oncology **6**(3), 347–359 (2012)
20. Virmani, A.K., Rathi, A., Sathyanarayana, U.G., Padar, A., Huang, C.X., Cunnigham, H.T., Farinas, A.J., Milchgrub, S., Euhus, D.M., Gilcrease, M., Herman, J., Minna, J.D., F., G.A.: Aberrant methylation of the adenomatous polyposis coli (APC) gene promoter 1A in breast and lung carcinomas. Clinical Cancer Research **7**(7), 1998–2004 (2001)
21. Jin, Z., Tamura, G., Tsuchiya, T., Sakata, K., Kashiwaba, M., Osakabe, M., Motoyama, T.: Adenomatous polyposis coli (APC) gene promoter hypermethylation in primary breast cancers. British Journal of Cancer **85**(1), 69–73 (2001)
22. Sarrió, D., Moreno-Bueno, G., Hardisson, D., Sánchez-Estévez, C., Guo, M., Herman, J.G., Gamallo, C., Esteller, M., Palacios, J.: Epigenetic and genetic alterations of APC and CDH1 genes in lobular breast cancer: relationships with abnormal E-cadherin and catenin expression and microsatellite instability. International Journal of Cancer **106**(2), 208–215 (2003)
23. Blondel, V.D., Guillaume, J.-L., Lambiotte, R., Lefebvre, E.: Fast unfolding of communities in large networks. Journal of statistical mechanics: theory and experiment **2008**(10), 10008 (2008)
24. Krämer, A., Green, J., *et al.*: Causal analysis approaches in ingenuity pathway analysis. Bioinformatics **30**(4), 523–530 (2013)
25. Shedden, K., Taylor, J.M.G., *et al.*: Gene expression–based survival prediction in lung adenocarcinoma: a multi-site, blinded validation study. Nature Medicine **14**(8), 822–827 (2008)
26. Dai, M., Lu, J.-J., *et al.*: BPTF promotes tumor growth and predicts poor prognosis in lung adenocarcinomas. Oncotarget **6**(32), 33878–33892 (2015)
27. Buganim, Y., Goldstein, I., Lipson, D., Milyavsky, M., Polak-Charcon, S., Mardoukh, C., Solomon, H., Kalo, E.,

Madar, S., Brosh, R., Perelman, M., Navon, R., Goldfinger, N., Barshack, I., Yakhini, Z., Rotter, V.: A novel translocation breakpoint within the BPTF gene is associated with a pre-malignant phenotype. PloS One **5**(3), 9657 (2010)
28. Grinberg-Rashi, H., Ofek, E., Perelman, M., Skarda, J., Yaron, P., Hajdúch, M., Jacob-Hirsch, J., Amariglio, N., Krupsky, M., Simansky, D.A., Ram, Z., Pfeffer, R., Galernter, I., Steinberg, D.M., Ben-Dov, I., Rechavi, G., Izraeli, S.: The expression of three genes in primary non–small cell lung cancer is associated with metastatic spread to the brain. Clinical Cancer Research **15**(5), 1755–1761 (2009)
29. Lu, Y., Wang, L., *et al.*: Gene-expression signature predicts postoperative recurrence in stage I non-small cell lung cancer patients. PloS One **7**(1), 30880 (2012)
30. Haferlach, T., Kohlmann, A., Wieczorek, L., Basso, G., Kronnie, G.T., Béné, M.-C., Vos, J.D., Hernández, J.M., Hofmann, W.-K., Mills, K.I., Gilkes, A., Chiaretti, S., Shurtleff, S.A., Kipps, T.J., Rassenti, L.Z., Yeoh, A.E., Papenhausen, P.R., Liu, W.-M., Williams, P.M., Foá, R.: Clinical utility of microarray-based gene expression profiling in the diagnosis and subclassification of leukemia: report from the international microarray innovations in leukemia study group. Journal of Clinical Oncology **28**(15), 2529–2537 (2010)
31. Yao, R., Feng, W.T., Xu, L.J., Zhong, X.M., Liu, H., Sun, Y., Zhou, L.L.: DUXAP10 regulates proliferation and apoptosis of chronic myeloid leukemia via PTEN pathway. European Review for Medical and Pharmacological Sciences **22**(15), 4934–4940 (2018)
32. Klemm, L., Duy, C., Iacobucci, I., Kuchen, S., von Levetzow, G., Feldhahn, N., Henke, N., Li, Z., Hoffmann, T.K., Kim, Y.-m., Hofmann, W.-K., Jumaa, H., Groffen, J., Heisterkamp, N., Martinelli, G., Lieber, M.R., Casellas, R., Müschen, M.: The B cell mutator AID promotes B lymphoid blast crisis and drug resistance in chronic myeloid leukemia. Cancer Cell **16**(3), 232–245 (2009)
33. Pizzatti, L., Panis, C., Lemos, G., Rocha, M., Cecchini, R., Souza, G.H., Abdelhay, E.: Label-free MSE proteomic analysis of chronic myeloid leukemia bone marrow plasma: disclosing new insights from therapy resistance. Proteomics **12**(17), 2618–2631 (2012)
34. Im Kim, K., Park, J., Ahn, K.-S., Won, N.-H., Kim, B.K., Shin, W.G., Yoon, S.-S., Oh, J.M.: Molecular characterization and prognostic significance of FLT3 in CML progression. Leukemia Research **34**(8), 995–1001 (2010)
35. Burguillo, F.J., Martin, J., Barrera, I., Bardsley, W.G.: Meta-analysis of microarray data: the case of imatinib resistance in chronic myelogenous leukemia. Computational Biology and Chemistry **34**(3), 184–192 (2010)
36. Puissant, A., Auberger, P.: AMPK-and p62/SQSTM1-dependent autophagy mediate resveratrol-induced cell death in chronic myelogenous leukemia. Autophagy **6**(5), 655–657 (2010)
37. Puissant, A., Dufies, M., Raynaud, S., Cassuto, J.P., Auberger, P.: Targeting lysosomes to eradicate imatinib-resistant chronic myelogenous leukemia cells. Leukemia **24**(5), 1099–1101 (2010)
38. Yamada, Y., Warren, A.J., Dobson, C., Forster, A., Pannell, R., Rabbitts, T.H.: The t cell leukemia lim protein lmo2 is necessary for adult mouse hematopoiesis. Proceedings of the National Academy of Sciences **95**(7), 3890–3895 (1998)
